# Low-Heating-Rate Thermal Degradation of Date Seed Powder and HDPE Plastic: Machine Learning CDNN, MLRM, and Thermokinetic Analysis

**DOI:** 10.3390/polym17060740

**Published:** 2025-03-11

**Authors:** Zaid Abdulhamid Alhulaybi Albin Zaid, Abdulrazak Jinadu Otaru

**Affiliations:** Department of Chemical Engineering, College of Engineering, King Faisal University, Al Ahsa 31982, Saudi Arabia

**Keywords:** date seed powder, high-density polyethylene, thermal degradation, machine learning, thermokinetics, circular economy

## Abstract

Finding reliable, sustainable, and economical methods for addressing the relentless increase in plastic production and the corresponding rise in plastic waste within terrestrial and marine environments has garnered significant attention from environmental organizations and policymakers worldwide. This study presents a comprehensive analysis of the low-heating-rate thermal degradation of high-density polyethylene (HDPE) plastic in conjunction with date seed powder (DSP), utilizing thermogravimetric analysis coupled with Fourier transform infrared spectroscopy (TGA/FTIR), machine learning convolutional deep neural networks (CDNNs), multiple linear regression model (MLRM) and thermokinetics. The TGA/FTIR experimental measurements indicated a synergistic interaction between the selected materials, facilitated by the presence of hemicellulose and cellulose in the DSP biomass. In contrast, the presence of lignin was found to hinder degradation at elevated temperatures. The application of machine learning CDNNs facilitated the formulation and training of learning algorithms, resulting in an optimized architectural composition comprising three hidden neurons and employing 27,456 epochs. This modeling approach generated predicted responses that are closely aligned with experimental results ($R^{2}\;\sim \;0.939$) when comparing the responses from a formulated MLRM model ($R^{2}\;\sim \;0.818$). The CDNN models were utilized to estimate interpolated thermograms, representing the limits of experimental variability and conditions, thereby highlighting temperature as the most sensitive parameter governing the degradation process. The Borchardt and Daniels (BD) model-fitting and Kissinger–Akahira–Sunose (KAS) model-free kinetic methods were employed to estimate the kinetic and thermodynamic parameters of the degradation process. This yielded activation energy estimates ranging from 40.419 to 91.010 kJ·mol⁻^1^ and from 96.316 to 226.286 kJ·mol⁻^1^ for the selected kinetic models, respectively, while the D2 and D3 diffusion models were identified as the preferred solid-state reaction models for the process. It is anticipated that this study will aid plastic manufacturers, environmental organizations, and policymakers in identifying energy-reducing pathways for the end-of-life thermal degradation of plastics.

## 1. Introduction

Plastics are primarily produced by the petrochemical industry [1], and they have a wide range of useful applications in both domestic and industrial contexts. Typical examples of these applications include food packaging, biomedical devices, automotive and aerospace components, decorative household and office items, computers, educational materials, and electrical and electronic appliances. Additionally, the emerging trends in three-dimensional (3D) printing have facilitated the extensive applications of plastics and fiber-reinforced polymer composites within the domain of additive manufacturing [2]. However, the heightened production of these materials and their subsequent end-of-life disposal in terrestrial and marine environments have led to widespread environmental pollution and climate change, exacerbated by issues of food contamination. In alignment with the Saudi Green Initiative and Sustainable Development Goals 13–15, there is an urgent need to develop more sustainable and cost-effective strategies for managing this persistent waste in the environment. Over the years, thermal degradation has been employed as a dependable method for converting plastics into ash [3,4]; however, this approach incurs significant energy costs associated with the successful execution of such operations. Consequently, there is a pressing need to explore alternative strategies that enhance the thermal degradation of polymers, particularly through the utilization of agro-waste as a substitutive material to help diminish the energy requirements necessary for the thermal degradation of plastics.

Analogous research studies documented in the literature concerning polymer degradation have predominantly concentrated on thermal stability, which frequently leads to the estimation of the corresponding reaction mechanisms, as well as kinetic and thermodynamic properties. For instance, Xu et al. [5] examined the thermal decomposition of polyvinyl chloride (PVC), polypropylene (PP), and low-density polyethylene (LDPE) by using a thermogravimetric analyzer (TGA) at the elevated heating rates of 100, 300, and 500 °C·min^−1^. Both the Criado master plots and Coats–Redfern model-fitting methods were employed to establish the resultant reaction mechanisms for the pyrolysis processes, while the activation energies were estimated by utilizing the Kissinger–Akahira–Sunose (KAS), Flynn–Wall–Ozawa (FWO), and Friedmann isoconversional model-free methods. In a related study conducted by Aboulkas et al. [6], the pyrolysis of high-density polyethylene (HDPE), polypropylene (PP), and low-density polyethylene (LDPE) was performed in a TGA at the four distinct heating rates of 2, 10, 20, and 50 °C·min^−1^ under inert nitrogen gas flow conditions. The study [6] identified the contracting cylinder model as the appropriate solid-state reaction mechanism for the pyrolysis of PP, while the contracting sphere model was determined to be suitable for the pyrolysis of HDPE and LDPE. Furthermore, activation energy values ranging from 179 to 188 kJ·mol⁻^1^, from 215 to 221 kJ·mol^−1^, and from 238 to 247 kJ·mol^−1^ were obtained for the thermal decomposition of PP, LDPE, and HDPE, respectively.

Recent studies in the literature have demonstrated that co-pyrolysis, which involves the incorporation of agricultural waste into plastic waste, is more advantageous than the conventional thermal decomposition of plastic waste alone. This approach has been shown to provide an energy reduction strategy, thereby facilitating effective waste management. For instance, Chen et al. [7] investigated the role of Paulownia wood (PW) in the thermal degradation of polyvinyl chloride (PVC), polyethylene (PE), and polypropylene (PP) by using a thermogravimetric analyzer (TGA). The study revealed a synergistic interaction during the co-pyrolysis of these plastics and biomass, as evidenced by a reduction in thermal stability, a decrease in activation energy, and an increase in residual ash content with higher biomass content in the composites. Albin Zaid and Otaru [8] identified the first-order reaction kinetics as suitable for describing the solid-state reaction mechanism during the co-pyrolysis of date seeds and polypropylene, conducted over a degradation temperature range between 25 °C and 600 °C and at the heating rates of 10, 20, and 40 °C·min^−1^. The study [8] estimated activation energies of 51.471 and 51.221 kJ·mol^−1^ by using the Coats–Redfern and general Arrhenius model-fitting methods, respectively, while values of 156.080 and 153.767 kJ·mol^−1^ were obtained through the application of the Flynn–Wall–Ozawa (FWO) and Kissinger–Akahira–Sunose (KAS) model-free methods. Dobrzyńska-Mizera et al. [9] indicated that increasing the composition of walnut shells (i.e., 10, 20, and 40 wt%) reduces the enthalpy changes for pure polypropylene from 110.2 J·g^−1^ to 100.2, 84.0, and 72.2 J·g^−1^, respectively. Părpăriţă et al. [10] concluded that the thermal behavior observed from the co-pyrolysis of various lignocellulosic materials (*Eucalyptus globulus*, Norway spruce, energy grass, *Brassica rapa*, pinecones, and grape seeds) with polypropylene plastic is contingent upon the cellulose, hemicellulose, lignin, and ash composition of the biomass. The resulting thermograms of the propylene blends in their study indicated a two-step degradation process, with shifts in the thermograms toward lower temperature minima as the biomass content increased.

Other studies related to the co-pyrolysis of biomass and waste plastics have been documented [11,12,13,14], with most of this work being dedicated to the synergistic interactions that occur between these materials, as well as the estimation of reaction mechanisms, kinetics, and thermodynamic parameters. Recently, investigations into the thermal decomposition of plastics have incorporated machine learning techniques to predict either weight loss during thermal degradation or the process parameters associated with thermogravimetric analysis (TGA) experimental measurements. For instance, Alhulaybi and Otaru [15] employed backpropagation deep neural networks, a machine learning technique, to train a series of pre-processed experimental datasets regarding sample weight loss obtained from the co-pyrolysis of *Phoenix dactylifera* L. and high-density polyethylene (HDPE) blends conducted at the heating rates of 10, 20, and 40 °C·min^−1^, with degradation temperatures ranging between 25 and 600 °C. The study revealed a coefficient of determination approaching unity between experimental measurements and predictions; the established model was subsequently used to project degradation temperatures between 600 and 1000 °C. In 2023, Potnuri et al. [16] demonstrated the applicability of machine learning support vector machines (SVMs) in predicting yields obtained from the microwave-assisted co-pyrolysis of biomass and plastics, estimating the coefficients of determination for biogas, biochar, and bio-oil yields as 0.91, 0.93, and 0.96, respectively. Zhang et al. [17] indicated that the gradient boosting decision tree algorithm provided the best predictions (R^2^~0.9) for monoaromatic-rich oil yield from the co-pyrolysis of biomass and waste plastic in the presence of a zeolite catalyst, compared with other machine learning models such as extreme gradient boosting, light gradient boosting, and random forest.

Dates are primarily cultivated in Saudi Arabia and other regions of the Middle East, resulting in a significant accumulation of date waste in the environment. Additionally, Saudi Arabia is home to numerous petrochemical and plastic manufacturing industries, whose operations have led to an ongoing increase in plastic production (low- and high-density polyethylene, polyethylene terephthalate, polystyrene, polyvinyl chloride, polyvinyl alcohol, polycarbonate, etc.) and associated environmental waste. Relevant research documented in the literature has demonstrated that various agro-derived wastes, including walnut shells, groundnut shells, coconut shells, and rice husks, have been employed to enhance the thermal stability of polymers. However, there exist a limited number of studies specifically focusing on low-heating-rate thermogravimetric analysis (TGA) operations and the application of machine learning tools in this context. Previous reports published by this research group have documented the utilization of DSP as a feedstock in the pyrolysis of polypropylene (PP) [8] and high-density polyethylene (HDPE) [15]. Both studies were conducted at the heating rates of 10, 20, and 40 °C.min⁻^1^. It is important to note that the PP grade employed in [8] is PP520L homopolymer, characterized by a density of 905 kg.m^−3^ and a melt flow rate (MFR) of 10 g/10 min at 230 °C and a load of 2.16 kg. The HDPE grade utilized in [15] is HDPE F00952 copolymer, characterized by a density of 952 kg.m^−3^, an MFR value of 0.05 dg/min at 190 °C, and a load of 2.16 kg. The differences in the physical and chemical properties of these two polymeric materials are reflected in their characteristic thermograms, which indicate that the onset of the propagation stage of thermal degradation for the HDPE materials begins at 365, 385, and 485 °C for the three heating rates, respectively [15]. In contrast, the propagation stage for the PP materials occurs within a lower temperature range of 325 to 495 °C, with minimal deviations observed in the thermograms obtained for these heating rates [8]. This study, therefore, presents a comprehensive analysis of the thermal degradation of date seed powder (DSP) and high-density polyethylene (HDPE) conducted in a TGA system at the low heating rates of 2, 5, and 10 °C·min^−1^, with degradation temperatures ranging from 25 to 600 °C. The analyses encompass TGA-FTIR experimental measurements, convolutional deep neural networks (CDNNs), multiple linear regression modeling, and the kinetics and thermodynamics of the process. The experimental component of this study is anticipated to elucidate the influence of low-heating dominant temperature effects on the pyrolysis and co-pyrolysis processes, in comparison with established literature data. Furthermore, the machine learning modeling and simulation aspect of this research study is expected to yield valuable insights into the accurate predictions of the thermogravimetric analysis (TGA) thermograms generated from the study, as well as to establish a hierarchical ordering of the significance of the operational parameters employed in the thermal degradation of the materials at these low heating rates.

## 2. Experimental Approach and Data

The experimental approach of this study entails the collection of dried date seed samples from Al Hofuf, Al Ahsa, located in the eastern region of Saudi Arabia, along with SABIC^®^ HDPE F00952 plastic produced by Saudi Basic Industries Corporation (SABIC). This specific HDPE plastic from SABIC is characterized by a density of 952 kg/m^3^ and melt flow rates (MFRs) of 0.05 g/10 min at 190 °C under a load of 2.16 kg and 9.0 g/10 min at 190 °C under a load of 21.6 kg and has been specifically designed for blown film extrusion [18]. Clean samples of the date seeds were oven-dried at a temperature of about 50 °C for 12 h, followed by crushing to obtain a powdered form characterized by particle sizes ranging from 100 to 200 µm. A similar crushing procedure was applied to the HDPE plastic prior to conducting thermogravimetric analysis and Fourier transform infrared (FTIR) measurements.

The Fourier transform infrared (FTIR) measurements were conducted by utilizing a Cary 630 FTIR system, which is equipped with MicroLab 5.8 software (Agilent Technologies, Santa Clara, CA, USA). The objective of this experimental measurement is to identify the molecular compounds present in both date seeds and high-density polyethylene (HDPE) plastic [19]. Acetone was employed to clean the crystal section of the FTIR system prior to placing the individual powder samples onto it. A pressing mechanism was used to secure the sample on the crystal before measuring the transmittance of infrared radiation across varying wavelengths for each sample. The pyrolysis and co-pyrolysis processes were executed by using a Mettler Toledo thermogravimetric analysis (TGA) system, following methodologies consistent with those described in [6,20,21]. Before initiating TGA operation, a nitrogen gas flow at a rate of 40 mL·min^−1^ was introduced into the system to maintain an oxidation-free environment, thereby preventing mass loss due to combustion. Pyrolysis processes were performed on individual samples, while co-pyrolysis studies involved synthetically created composite mixtures of these samples, incorporating 25 and 50 wt% date seed powder (DSP) compositions. TGA was conducted across degradation temperatures ranging from 25 to 600 °C, employing varying low heating rates of 2, 5, and 10 °C·min^−1^. During TGA operation, the mass loss profile (thermogram) of individual samples and their blends was recorded in relation to degradation temperatures of up to 600 °C. This maximum temperature was selected as complete decomposition to ash is anticipated for the HDPE plastic material, which is the primary focus of degradation, as well as for most plastic materials. Upon reaching this maximum temperature, the TGA system was deactivated and allowed to cool before we recorded the final weight of the sample. A total of twelve TGA experiments were conducted on both pure and blended samples across the three selected low heating rates: 2, 5, and 10 °C·min^−1^.

**Figure 1:**
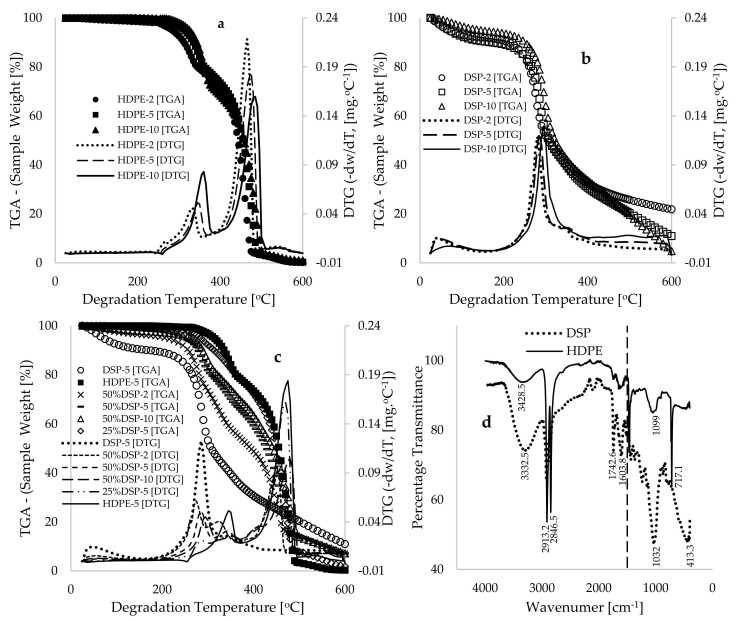
Plots of TGA sample weight [%] and DTG data [mg.°C^−1^] conducted at heating rates of 2, 5, and 10 °C.min^−1^ plotted against degradation temperature for (**a**) HDPE, (**b**) DSP, and (**c**) DSP/HDPE blends. (**d**) FTIR data for DSP and HDPE, demonstrating percentage transmittance against wavenumber [cm^−1^].

Figure 1a presents the thermogravimetric (TGA) and derivative thermogravimetric (DTG) for the HDPE material, generated at the heating rates of 2, 5, and 10 °C·min^−1^ against degradation temperatures ranging from 25 to 600 °C. The thermograms in Figure 1a indicate slight shifts in the temperature maxima with the increase in the heating rate. Each resulting thermogram can be categorized into initiation, propagation, and termination stages. Specifically, Figure 1a illustrates that both the initiation and termination stages of degradation occur at 25–280 °C and 495–600 °C, respectively. Furthermore, Scott [22] describes the initiation of polymer degradation as the phase characterized by either the formation of free radicals through the direct reaction of oxygen with the polymer or the homolysis of C–C or C–H bonds within the molecules. According to a report in [23], the initiation stage in polymer degradation is largely characterized by the loss of water molecules, which accounts for less than 1.0 wt% weight loss in the sample during this stage of HDPE thermal degradation, as shown in Figure 1a. Lamar et al. [23] reported that the moisture content of HDPE is 0.045 wt%, with values ranging between 0.01 and 0.30 wt%, noted in [24] as the water absorption capacity for this material. In contrast, the termination stage of HDPE degradation can be referred to as its complete degradation to ash, resulting in over 95 wt% loss from the original sample, as evidenced in Figure 1a.

Figure 1a illustrates that a significant amount of weight loss (exceeding 90 wt%) was observed for all the HDPE thermograms, which occurred within degradation temperatures ranging from 280 to 495 °C. Both the TGA and DTG curves indicate a two-step degradation process during this propagation stage of pyrolysis and co-pyrolysis processes. For example, at a heating rate of 2 °C.min^−1^, the DTG curves reveal two distinct peaks at 335 °C and 365 °C, while the thermogram conducted at a heating rate of 10 °C.min^−1^ yielded peak values of 360 °C and 485 °C. The DTG peaks associated with HDPE degradation not only signify the degradation steps but also indicate the temperatures at which the most significant chemical reactions and conversions occur. Figure 1d presents the FTIR data, showcasing percentage transmittance as a function of wavenumber (cm^−1^) for both HDPE and DSP samples. In the case of the HDPE sample, five distinct peaks were recorded at 3428.5, 2913.2, 2846.5, 1099, and 717.1 cm^−1^. According to the FTIR database referenced in [25], the broad peak at 3428.5 cm^−1^ falls within the range of 3200–3550 cm^−1^ and can be attributed to the O–H stretching of alcohol, while the sharp peaks at 2913 cm^−1^ and 2846 cm⁻^1^ correspond to the C-H stretching of alkanes. The peaks at 1099 cm^−1^ and 717 cm⁻^1^ can be identified as the C–O stretching of secondary alcohol and the C=C bending of alkenes, respectively; however, both fall within the fingerprint region (<1500 cm^−1^ [26]), which has been widely reported as a less reliable area for molecular identification [27]. Consequently, the propagation stage of HDPE plastic degradation, as illustrated in Figure 1a, can be characterized as the region of complete degradation of these compositions, resulting from the scission of chemical bonds and the release of free radicals [28]; the rapid depolymerization of larger molecules into smaller ones [29,30]; the formation of amorphous, semi-crystalline, and crystalline phases [23]; and a reduction in the apparent density of the sample with the increase in degradation temperature.

Figure 1b presents TGA-DTG data for the pyrolysis and co-pyrolysis of the DSP biomass material conducted at the heating rates of 2, 5, and 10 °C.min^−1^. Analogous to the degradation of HDPE, the characteristic trends of the DSP biomass shifted towards higher temperature maxima with the increase in the heating rate. Nonetheless, the variation in degradation trends can be attributed to the composition of the DSP biomass. The FTIR analysis of the DSP biomass, as depicted in Figure 1d, reveals four significant peaks in the functional group region. The broad peak observed at 3332.5 cm⁻^1^ falls within the 3200–3550 cm^−1^ range and can be ascribed to O–H stretching associated with alcohol. The sharp peaks at 2921.6 cm^−1^, 1742.6 cm^−1^, and 1603.2 cm^−1^ are classified as the O–H stretching of cellulose and hemicellulose, the C=O stretching of ∂-lactone (indicative of pectin and hemicellulose), and the C=C stretching of lignin, respectively. The TGA data presented in Figure 1b indicate that the initiation stage of pyrolysis for DSP biomass occurs within a degradation temperature range of 25 to 225 °C, resulting in an approximate material loss of 9 wt%. In contrast to the thermal degradation of HDPE, this considerable weight loss can be ascribed to the elevated moisture content characteristic of DSP biomass. An experimental study on the physicochemical properties of date seed powder [31] reported moisture content values ranging from 4.76 to 8.02 wt% for date seeds from Saudi Arabia. In a related investigation, Himanshu et al. [32] identified a moisture content of 4.34 wt%, while another study documented values of 4.60 and 8.30 wt% for date seeds sourced from various regions in Morocco [33]. Consequently, the data illustrated in Figure 1b demonstrate that the decomposition of cellulose and hemicellulose within the DSP biomass occurs between 225 and 400 °C. Approximately 55 to 60 wt% of the sample is lost during this primary reaction stage, as evidenced by the DTG peaks observed at temperatures ranging from 285 to 290 °C, as depicted in Figure 1b. Beyond this stage, Figure 1b indicates that gradual degradation (slow) of the lignin component occurs during the thermogram performed at a heating rate of 10 °C.min^−1^, while those conducted at 2 and 5 °C.min^−1^ ultimately degrade to ash at a maximum temperature of 600 °C.

Figure 1c presents TGA thermograms and DTG curves for the DSP, 50%DSP, 25%DSP, and HDPE samples, all obtained at a constant heating rate of 5 °C·min^−1^. This figure indicates a shift in temperature maxima with the increase in the proportion of DSP in the blend. For instance, at a sample weight of 60 wt%, degradation temperature values of 292, 395, 435, and 460 °C were observed for the DSP, 50%DSP, 25%DSP, and HDPE samples, respectively. This trend is consistent with previous reports involving the co-pyrolysis of walnut shell and polypropylene (PP) [9], lignocellulosic biomass and plastics [12], and lychee seed and plastic waste [34]. The observed decrease in thermogram temperature with the increase in the DSP proportion in the blends may be attributed to the low thermal conductivity [35] and high moisture content [33] of DSP biomass. Consequently, this results in reduced thermal stability of the composite when compared with the pyrolysis of pure HDPE plastic material. Furthermore, the DTG curves in Figure 1c indicate multi-step degradation during the propagation stage of the degradation process, which can be attributed first to the decomposition of hemicellulose and cellulose components in the DSP biomass, followed by the decomposition of the HDPE plastic and lignin contents of the DSP material. In summary, both the TGA and DTG curves presented in Figure 1c indicate that the presence of hemicellulose and cellulose in the DSP biomass contributes to the reduced thermal stability of HDPE plastic blends under the specified operational conditions of heating rates (2, 5, and 10 °C·min^−1^) and degradation temperatures (25–600 °C). Both cellulose and hemicellulose are hydrolyzed by extracellular enzymes, resulting in higher affinity for water compared with lignin, rendering them more susceptible to early thermal degradation [36]. Conversely, lignin decomposition, as discussed in [5], is regarded as a rate-limiting step in biomass degradation due to the complexity of its bonds, cross-linkages, and low nitrogen content [37]. Furthermore, Lv et al. [38] reported a significant reduction in mass during cellulose volatilization in the pyrolysis process, as well as gradual mass degradation that plateaued, which was attributed to the degradation of the lignin component. Moreover, the resulting thermograms for the DSP materials align with findings in [39], which suggest that the rate of pyrolysis of biomass is directly proportional to the cellulose fractions and inversely proportional to the lignin fractions present in the biomass.

## 3. Machine Learning CDNN Approach and Analysis of Data

Figure 1 illustrates that the TGA experimental data for individual samples and their blends exhibit significant variability, which can be attributed to differences in heating rates, degradation temperatures, degradation times, and sample compositions. Consequently, identifying a model that accurately describes all experimental data points may be challenging. Instead, obtaining a precise function that captures the general trends of the experimental data points would be a more suitable strategy. In this study, machine learning convolutional deep neural network (CDNN) modeling techniques were employed to formulate the algorithms necessary for predicting the mass profile loss (thermogram) associated with pyrolysis and co-pyrolysis processes. This process involved identifying operating parameters, classifying them into features (inputs) and labels (outputs), and establishing the relationship between them prior to data pre-processing. The label for this modeling approach is the mass profile loss obtained from the process, which can be considered the dependent variable, while the independent variables (features) include the heating rate (*Q*), degradation temperatures (*T*), degradation time (*t*), and the fractional composition of date seed powder in the composite (*C*).

The data pre-processing of the raw experimental data was conducted to identify the most significant data points for modeling and simulation. A total of 110,408 data points per variable were measured experimentally. The number of experimental data points and the timing of the TGA experiment depend on the heating rate employed during the operation, with each data point recorded every second. For instance, a total of 17,250 data points per variable were acquired for TGA experiments conducted at a heating rate of 2 °C.min^−1^, across degradation temperatures ranging from 25 to 600 °C. A total of 6900 and 3450 data points were obtained for samples tested at the heating rates of 5 °C.min^−1^ and 10 °C.min^−1^, respectively. Considering this, data point selection was performed by selecting features and labels obtained at every 10 °C interval for degradation temperatures between 25 and 255 °C (primarily data from the initiation stage) and 500 to 600 °C (primarily data from the termination stage). Additionally, an interval of 5 °C was utilized for data point selection within the degradation temperatures of 255 to 500 °C, as this range represents the most critical phase of the degradation process (primarily data from the propagation stage), where the dominant reaction processes are expected to occur, as evidenced by the DTG curves in Figure 1. By using this approach, a total of 966 data points per variable were selected for log normalization before being used as input for the model. The log normalization of the selected experimental data points was achieved by scaling the selected features and label values to a range between 0 and 1. This was accomplished by dividing individual data points by a chosen maximum value that is realistically possible [8,40]. For instance, the heating rates (*Q*) were divided by 100 °C·min⁻^1^, while the degradation temperature (*T*), degradation time (*t*), and DSP composition (*C*) were divided by 1000 °C ($T_{max}$), 20,000 s (t), and ($C_{max}$), respectively. This approach is essential to converting the data into dimensionless values and is integral to the selection of the Sigmoid activation function for the deep learning training of the experimental datasets, which will be discussed later.

**Figure 2:**
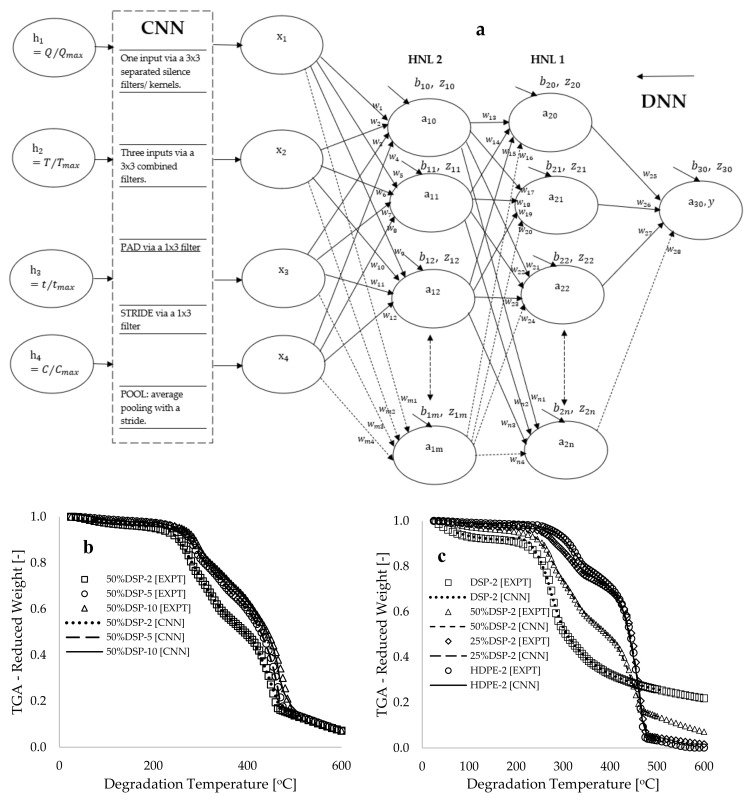
(**a**) A CDNN framework showing typical input, hidden, and output neurons, as well as a comparison of experimental and CNN-predicted values of reduced weight [-] against the degradation temperature [°C] for (**b**) 50% sample composition at different heating rates and (**c**) varying sample compositions at a constant heating rate of 2 °C.min^−1^.

Figure 2a presents a convolutional deep neural network (CDNN) framework that illustrates the connection between input data and the convolutional neural network (CNN) segment of the framework, the output from the CNN to the deep neural network (DNN) component, the hidden neurons within the DNN framework, and the output neuron. The CNN segment depicted in Figure 2a demonstrates the transmission of selected features and labels through a series of kernels (filters), which function as data extractors to produce a convolutional output referred to as a vector [41]. The filter is commonly employed in convolutional operations to detect silence at any position within the data, and its operations can be conceptualized as a small multilayer perceptron (MLP) that traverses the input signal [42], thereby inducing a temporal shift in the output signal. In Figure 2a, the selected input data are processed through three 3 × 3 separated filters containing values of −1 and +1, resulting in the generation of three distinct vectors. These multiple vectors are subsequently subjected to another combined 3 × 3 filter, yielding a single vector. To preserve the classification of dimensionality, a one-dimensional padding was applied to both boundaries, followed by the passage of the vector through a 1 × 3 filter. A two-step convolutional stride and an average pooling operation were performed on the vector to produce the final reduced classification. The output data obtained from the convolution operator are represented as a single-column vector with values ranging between 0 and 1.0. A total of 420 data points per variable were extracted from the 996 selected data points following the convolutional operation. Figure 2b,c illustrate plots of selected experimental data (EXPT) and the CNN-processed data against the degradation temperature. These figures display complete overlaps between the CNN-modeled and selected experimental data (R^2^~0.999), indicating that the convolutional kernels have been effectively learned. Therefore, it is essential to recognize that this convolutional approach may be characterized as a data selection technique—extracting significant features and labels for modeling while reserving the unselected data for cross-validation (to be discussed further).

Following convolutional neural network (CNN) modeling, the deep neural network (DNN) segment of the framework illustrated in Figure 2a was implemented by constructing an architecture comprising ten hidden neurons, distributed equally across two hidden layers, as depicted in Figure S1 of the Supplementary Materials. The general artificial neural network (ANN) models utilized for the formulation of learning algorithms are presented in Equations (1)–(3). Equation (1) conveys a linear representation of the sum weight (Z) as a function of the synaptic weight ($w_{i}$), bias ($b_{i}$), and input function ($x_{i}$). Equation (2) represents the Sigmoid activation function, while Equation (3) delineates the loss or cost function, which indicates the square of the difference between the actual output (y) and the predicted output or activation (a). It is essential to emphasize that the variable included in the linear expression in Equation (1) facilitated the integration of the input parameters (heating rate, degradation temperature, degradation time, and sample composition) into the model developed according to the architecture presented in Figure 2a. Furthermore, Equation (3) is utilized to integrate the output experimental data of the fractional weight (y) obtained from the TGA measurements, which is necessary to compute the loss function for each activation value computed during the training of the formulated gradient descent algorithm (loss optimization function) derived for the framework illustrated in Figure 2a.

(1) $$Z=\sum_{i}w_{i}.x_{i}+b_{i}$$

(2) $$a=\sigma ’\left[z\right]=\frac{1}{1+e^{-z}}$$

(3) $$C={\left(y-a\right)}^{2}$$

**Figure 3:**
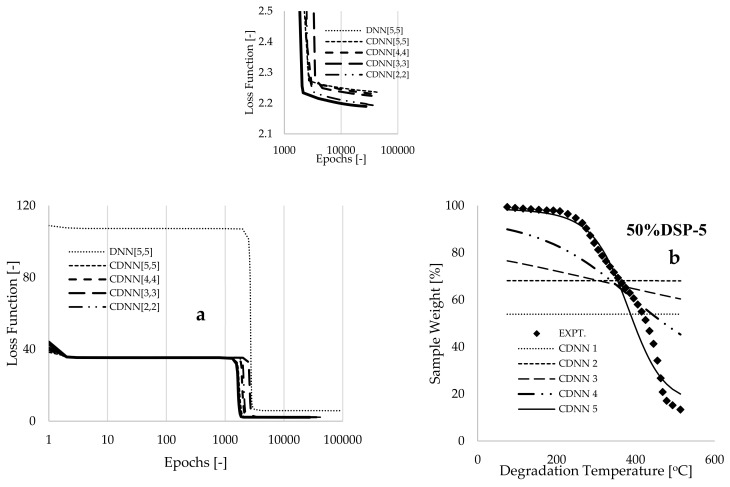

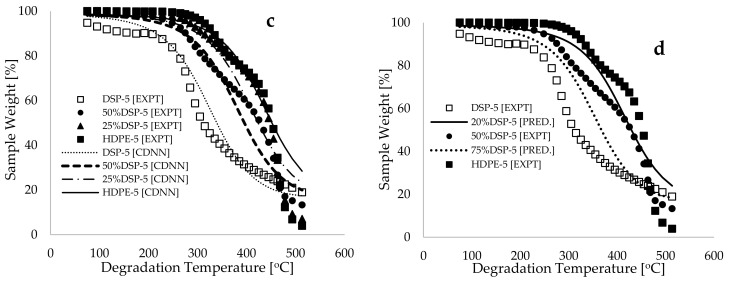
(**a**) Plots depicting the loss function for various modeling modifications over epochs and plots of experimentally measured versus predicted data for the (**b**) 50%DSP-5 sample, (**c**) varied sample compositions with a constant heating rate of 5 °C.min^−1^, and (**d**) interpolated data for 50%DSP and 75%DSP sample compositions versus degradation temperature [°C].

A comprehensive formulation of the learning algorithms, along with the cost (loss) optimization functions developed through the backpropagation method, was achieved by utilizing a procedure analogous to that reported in [43], as illustrated in the Supplementary Materials. Furthermore, the machine learning training was conducted by utilizing Microsoft Excel, complemented by a written looping script in Visual Basic for Applications, as detailed in the Supplementary Materials. At the commencement of training, an initial value of 0.1 was assigned to each of the synaptic weights and biases, and a computational time of one second was selected to complete each iteration of the loop. Subsequently, the loop time was accelerated to zero seconds to enhance modeling speed while ensuring that convergence was achieved. The objective of this approach was to identify the optimized synaptic weights and biases that significantly minimize the loss function to a value approaching zero, as this condition indicates a potential point of overlap between modeling and experimental data [44]. With an initial learning rate set to 5.0, Figure 3a demonstrates that the output from the DNN [5,5] framework, utilizing the chosen experimental datasets as input, produced a loss function of 108.844. Following the training processes, which encompassed approximately 97,044 epochs (iterations) over a span of 4.19 h, this loss function decreased to 5.866. When employing CNN-processed data as input features within the CDNN [5,5] framework, a substantial reduction in the loss function (38.428) was observed at the onset of training, as depicted in Figure 3a. Ultimately, this loss function of 38.428 was further reduced to 2.235 by the conclusion of the training process, utilizing a smaller number of epochs (45,655) and a training duration of 1.427 h.

During the training phase, hyperparameter modeling modifications were implemented by adjusting the learning rate from 5 to 2. This approach facilitated the convergence of the gradient descent algorithm (the formulated cost optimization algorithms), thus mitigating the issues associated with gradient explosion [45]. Additionally, this strategy contributed to a reduction in the training time and the number of epochs required for a significant decrease in the loss function to nearly zero. Figure 3a illustrates that further hyperparameter modeling was conducted through the systematic reduction in hidden neurons, followed by the training process. This method yielded some modeling gains, decreasing the loss function to 2.190 for a computational setup involving only three hidden neurons (i.e., the CDNN [2,1] framework), which utilized approximately 27,456 epochs and was computed over the course of one hour. Furthermore, model regularization was achieved by extending the training duration for the learning process to 72 h, resulting in a slight reduction in the loss function of the CDNN [2,1] framework to 2.153. This outcome corresponds to an approximate precision error of 1.697% when compared with the earlier loss function computed over one hour. It is essential to note that modeling regularization is crucial to addressing modeling complexity, as it facilitates a balance between underfitting and overfitting, thereby enhancing the model’s capability to learn more generalized patterns [41].

The validation and cross-validation of the models were accomplished by comparing the modeling results with experimental data and conducting interpolated predictions. Figure 3b illustrates the five progressive outputs of the Computational Deep Neural Network (CDNN) that were selected at various loss functions throughout the training process. CDNN 1 signifies the initiation of the training process, during which a constant value of 0.1 was assigned to all synaptic weights and biases, accompanied by a learning rate of 5.0 for the computational algorithms formulated within the CDNN [2,1] framework. The selection of these constants yielded a loss function value of 44.089, which subsequently decreased during the training phase, characterized by a series of modeling modifications. Figure 3b demonstrates a nearly complete overlap between the final predictive output of CDNN 5, represented as sample weight (%), and the experimental data for the 50%DSP sample assessed at a heating rate of 5 °C.min^−1^. Table 1 presents estimated uncertainties of 1.418 and 1.365 for the experimental and CDNN-predicted data, respectively. Furthermore, values of 4.906 and 0.165 were calculated as the mean absolute error (MAE), or L1 loss, and the mean bias error (MBE), respectively, while a coefficient of determination (R^2^) of 0.939 was obtained when comparing experimental data with the modeling response.

Figure 3c illustrates a substantial correlation between the modeling and experimental data regarding sample weight loss as a function of degradation temperature, specifically for samples of varied compositions subjected to a constant heating rate of 5 °C min^−1^. Figure 3d presents interpolated predictions for compositions of 20% and 75% DSP within a composite, indicating that these estimated predictions fall within the range of experimental variability. This observation suggests that the developed learning algorithms have successfully identified a more generalized pattern for predicting thermograms relevant to this process. Table 1 also presents the estimated final trained data of synaptic weights and biases, which were obtained following a series of modeling modifications that involved the elimination of certain hidden neurons, along with their associated synaptic weights and biases. The data in Table 1 indicate that the highest estimated value is for the connecting synaptic weight that links temperature (see Figure S1 in the Supplementary Materials) to the hidden neurons ($w_{2}=7.205$ and $w_{6}=7.205$). Thus, these estimated synaptic weight values in Table 1 show that the decreasing order of sensitivity of the operating parameters for this process is degradation temperature, sample composition, degradation time, and heating rate. It is essential to note that adjustments to the bias during the training process are beneficial to aligning the modeling outcomes with experimental data [44].

## 4. Comparison with Multiple Linear Regression Model (MLRM)

Considering Figure 2a, the mathematical expression for the multiple linear regression model (MLRM) delineating the relationship between the dimensionless inputs ($x_{1},\;x_{2},\;x_{3},\;and\;x_{4}$) and the output function (y) or ground truth (experimental value), is represented by Equation (4) below [46].

(4) $$y=a_{0}+a_{1}x_{1}+a_{2}x_{2}+a_{3}x_{3}+a_{4}x_{4}+e$$

(5) $$y_{p}=a_{0}+a_{1}x_{1}+a_{2}x_{2}+a_{3}x_{3}+a_{4}x_{4}$$

where $a_{0},a_{1},a_{2},a_{3},and\;a_{4}$ are empirical constants that, when estimated, could provide an alternative method for the prediction of the fractions of the sample weight (y) of the materials undergoing thermal degradation. The residual error is denoted by e, while $y_{p}$ represents the predicted response. Minimizing the sum of the squares of the residuals between the experimentally measured values (y) and predicted ($y_{p}$) values is crucial to the estimation of the empirical constants. This can be accomplished by taking the derivative of the square of the residual error (Equation (6)) to derive the least-squares fit.

(6) $$S_{r}=\sum_{i=1}^{n}e^{2}=\sum_{i=1}^{n}{\left(y-a_{0}-a_{1}x_{1}-a_{2}x_{2}-a_{3}x_{3}-a_{4}x_{4}\right)}^{2}$$

Therefore, rearranging the derivative of the square of the residuals with respect to each of the coefficients, as exemplified in [46], yields the set of normal equations illustrated in the matrix below, where n represents the number of data points for each variable and the values of the empirical constants obtained from the solution of the matrix by using the selected experimental data are $a_{0}=1.3516,\;a_{1}=0.3194,\;a_{2}=-1.9385,\;a_{3}=-0.0564,\;and\;a_{4}=-0.1745$. Consequently, the multiple linear regression model (MLRM) for predicting the fractional weight lost by the sample during the thermal degradation process is

(7) $$y_{p}=1.3516+0.3194x_{1}-1.9385x_{2}-0.0564x_{3}-0.1745x_{4}$$

$\sum_{i=1}^{n}y$
$a_{0}$n$\sum_{i=1}^{n}x_{1}$$\sum_{i=1}^{n}x_{2}$$\sum_{i=1}^{n}x_{3}$$\sum_{i=1}^{n}x_{4}$$\sum_{i=1}^{n}x_{1}.y$
$a_{1}$$\sum_{i=1}^{n}x_{1}$$\sum_{i=1}^{n}{x_{1}}^{2}$$\sum_{i=1}^{n}x_{1}.x_{2}$$\sum_{i=1}^{n}x_{1}.x_{3}$$\sum_{i=1}^{n}x_{1}.x_{4}$$\sum_{i=1}^{n}x_{2}.y$=$a_{2}$$\sum_{i=1}^{n}x_{2}$$\sum_{i=1}^{n}x_{2}.x_{1}$$\sum_{i=1}^{n}{x_{2}}^{2}$$\sum_{i=1}^{n}x_{2}.x_{3}$$\sum_{i=1}^{n}x_{2}.x_{4}$$\sum_{i=1}^{n}x_{3}.y$
$a_{3}$$\sum_{i=1}^{n}x_{3}$$\sum_{i=1}^{n}x_{3}.x_{1}$$\sum_{i=1}^{n}x_{3}.x_{2}$$\sum_{i=1}^{n}{x_{3}}^{2}$$\sum_{i=1}^{n}x_{3}.x_{4}$$\sum_{i=1}^{n}x_{4}.y$
$a_{4}$$\sum_{i=1}^{n}x_{4}$$\sum_{i=1}^{n}x_{4}.x_{1}$$\sum_{i=1}^{n}x_{4}.x_{2}$$\sum_{i=1}^{n}x_{4}.x_{3}$$\sum_{i=1}^{n}{x_{4}}^{2}$

**Figure 4:**
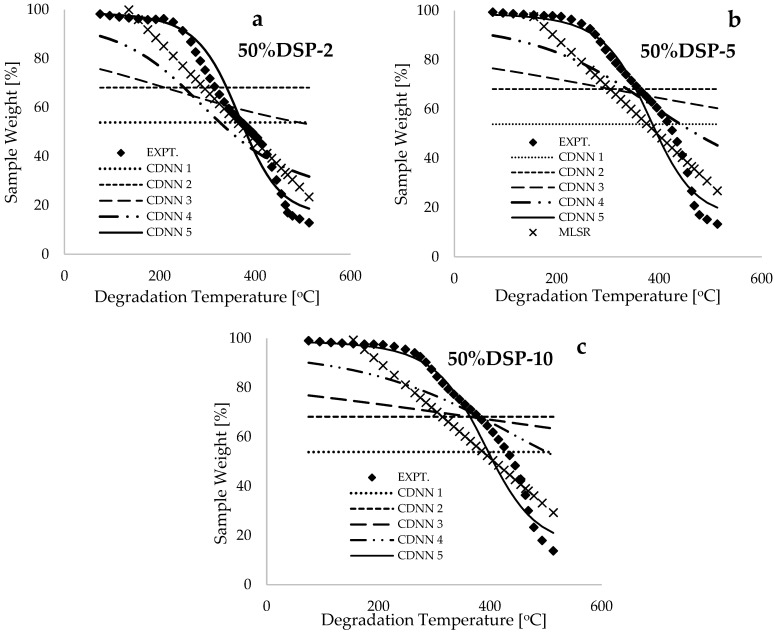
Plots of the experimentally measured and predicted (CDNN and MLSR) values of sample weight against degradation temperature for the (**a**) 50%DSP-2, (**b**) 50%DSP-5, and (**c**) 50%DSP-10 samples.

Figure 4 presents a comparison of the predicted weight loss (%) using the trained convolutional deep neural network (CDNN) model and the mathematically expressed multiple linear regression model (MLRM) for the 50% DSP blended samples at different low heating rates of 2, 5, and 10 °C.min^−1^. These plots indicate that the multiple linear regression model provided a poor prediction ($R^{2}\sim 0.812$) of the experimental measurements when compared with the predictions derived from the CDNN model ($R^{2}\sim 0.934$). This observation suggests that the rigorous training undertaken by the gradient descent algorithms, as formulated in the framework depicted in Figure 2a, resulted in the acquisition of more precise synaptic weights and biases, thereby enabling a more accurate prediction of the non-linear descriptive patterns observed at both the initiation and termination stages of the resulting experimental thermograms.

## 5. Thermokinetics

The thermokinetics of the pyrolysis and co-pyrolysis processes were investigated through the estimation of the kinetic and thermodynamic parameters associated with the process. The primary kinetic parameters of interest include the activation energy ($E_{A}$) and the pre-exponential factor (A). These parameters were estimated by utilizing both the Borchardt and Daniels (DB) model-fitting method for single heating rates (as expressed in Equation (8)) and the non-isothermal Kissinger–Akahira–Sunose (KAS) model-free method for variable heating rates (as indicated in Equation (9)). The selection of the BD and KAS kinetic models is intended to facilitate a comparative analysis of the estimated kinetic and thermodynamic data derived from both the single-heating-rate (BD) and multiple-heating-rate (KAS) models, respectively. A study referenced in [47] indicated that the application of multiple heating rates—specifically, a minimum of three—within model-free kinetic methods significantly enhances the reliability of representing non-isothermal data related to the pyrolysis and co-pyrolysis of materials. Nonetheless, the utilization of the BD model-fitting kinetic approach will support the estimation of potential reaction mechanisms, in addition to kinetic and thermodynamic data, based on a single heating rate [15]. While several alternative model-free methods exist—including the Flynn–Wall–Ozawa (FWO), Starink (STK), and Friedmann (FR) methods—there is documented evidence [4,5,6,8] indicating that these methods yield minimal significant variation in the estimated activation energy, which is a crucial kinetic parameter. Therefore, the employment of the BD and KAS models for the estimation of the kinetic and thermodynamic parameters in this study is justified. Table 2 presents the selected solid-state reaction mechanisms [6,48] that were employed alongside the BD model to infer the potential reaction mechanisms. Equations (10)–(12) provide mathematical expressions for estimating the thermodynamic parameters, specifically the changes in enthalpy (∆H), entropy (∆S), and Gibbs free energy (∆G) associated with the process [8,49]. In these equations, the term $x_{i}$ denotes the fractional conversion based on the sample weight percent, where $m_{0}$ represents the initial weight, $m_{t}$ is the weight at time t, and $m_{f}$ signifies the final weight of the sample. Additionally, the activation energy is measured in kJ·mol^−1^, R denotes the gas constant in J·mol^−1^·K^−1^, and $Q_{R}$ represents the heating rate (°C·min^−1^). Furthermore, $h=6.626\times 10^{-34}J.s^{-1}$ symbolizes Planck’s constant, K = 1.3806 × 10^−23^ J.K^−1^ is the Boltzmann constant, and T signifies the final or maximum temperature of constant conversion.

Borchardt and Daniels (BD) Model-Fitting Method for Single Heating Rates

(8) $$\ln\left[dx_{i}/dt\right]-n.[ln\left(1-x_{i}\right)]=\ln\left[\frac{g(x_{i})}{t}\right]=\ln A-\left(\frac{E_{A}}{R}\right).\frac{1}{T}$$

Kissinger–Akahira–Sunose (KAS) Model-Free Method for Variable Heating Rates

(9) $$ln[Q_{R}/T^{2}]=\ln\left(\frac{A.R}{E_{A}.\left(g\left(x_{i}\right)\right)}\right)-\left(\frac{E_{A}}{R}\right).\frac{1}{T}$$

Thermodynamic Properties

(10) $$∆H=E_{A}-R.T_{M}\;\;\;(\mathrm{Change}\;\mathrm{in}\;\mathrm{Enthalpy})$$

(11) $$∆S=R.\ln\left(\frac{A.h}{K.T_{M}}\right)\;\;(\mathrm{Change}\;\mathrm{in}\;\mathrm{Entropy})$$

(12) $$∆G=∆H-T_{M}.∆S\;\;\;(\mathrm{Change}\;\mathrm{in}\;\mathrm{Gibbs}\;\mathrm{Free}\;\mathrm{Energy})$$

Before the application of the BD and KAS kinetic models, it is essential to emphasize that the complete degradation of the HDPE plastic materials to ash was achieved at temperatures below 500 °C for the selected low heating rates (2, 5, and 10 °C·min^−1^). While the primary objective of this study is the complete degradation of plastic materials, the necessity to minimize energy requirements for this process facilitated the selection of the DSP biomass. Therefore, it is imperative to consider temperature and conversions within the confines of complete HDPE degradation for the kinetic and thermodynamic studies. The DTG peaks illustrated in Figure 1 were recorded below 500 °C, indicating that the synergistic interactions involving the degradation of the hemicellulose and cellulose contents in the biomass, as well as the degradation of the plastics, occurred prior to reaching this temperature. In other words, the principal reactions took place below this threshold. Furthermore, Figure 1b indicates that the presence of lignin in the DSP biomass retards degradation at elevated temperatures for the thermograms recorded at a heating rate of 10 °C·min^−1^. Notably, such a thermogram plateaued when compared with those obtained at the heating rates of 2 and 5 °C·min^−1^. Considering this, selecting data on mass loss for conversions below 20 wt% may overlook significant information from the thermogram at a heating rate of 10 °C·min^−1^. According to Vyazovkin et al. [50], the minimum number of thermograms required for the application of model-free isoconversional methods in estimating kinetic parameters is established. Thus, the selected BD and KAS kinetic models in this study were employed to estimate kinetic and thermodynamic parameters by using conversions ranging from 5 to 70 wt%.

**Figure 5:**
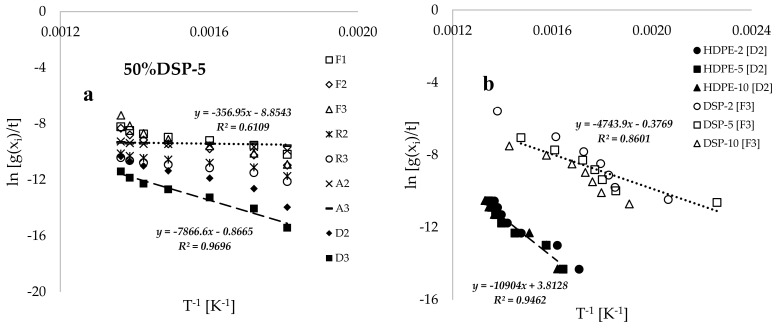

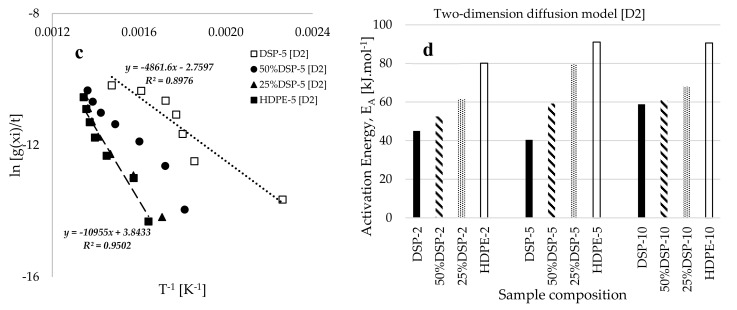
Plots of Borchardt and Daniels’ $\ln\left[g(x_{i})/t\right]$ of selected solid-state reaction mechanisms against the inverse of conversion temperature (T^−1^ [K^−1^]) for the (**a**) 50% DS-5 sample, (**b**) HDPE and DSP at different heating rates, and (**c**) samples of varied compositions and a constant heating rate of 5 °C.min^−1^ and (**d**) plots of estimated values of activation energy based on the two-dimension model [D2] against sample composition.

Figure 5a presents plots of Borchardt and Daniels’ ln(g($x_{i}$)/t) versus the inverse of conversion temperature (T^−1^, [K^−1^]) for the 50%DSP-5 sample, corresponding to the selected solid-state reaction mechanisms. The estimated values of kinetic and thermodynamic data by using the BD method are provided in Table 3. Both Table 3 and Figure 4a indicate that higher values of the coefficient of determination (R^2^) were achieved by using the D2 and D3 diffusion models, while poorer fits were obtained by using the A2 and A3 Avarami–Erofeev models. Figure 5b displays the plots of BD’s ln(g($x_{i}$)/t) against the inverse of conversion temperature (T^−1^, [K^−1^]) for the pure HDPE and DSP samples at various heating rates. This figure demonstrates that the trends associated with pure HDPE shifted towards the temperature maxima when compared with the linear inverse trends associated with pure DSP biomass, indicating a higher thermal stability of the HDPE plastic material. This finding is consistent with the study conducted in [9] on the thermal degradation of polyethylene plastic and walnut biomass. Additionally, Figure 5b illustrates that increasing the heating rate consistently results in a shift in the inverse linear trends towards the temperature maxima. However, these trends do not vary significantly due to the proximity of the selected heating-rate values when compared to the thermal degradation processes recorded for high heating rates in [6,11,15].

Figure 5c presents the characteristic plots of BD’s ln(g($x_{i}$)/t) against the inverse of conversion temperature (T^−1^, K^−1^) for samples with varying compositions and a constant heating rate of 5 °C·min^−1^. The data indicate that an increase in the composition of DSP in the blend consistently shifts the trends to lower temperatures, thereby reducing the thermal stability of the blended sample. Figure 5d illustrates the activation energies ($E_{A}$) obtained for the twelve samples created at different sample compositions and heating rates. The results demonstrate that the activation energy consistently increases with a higher HDPE composition in the composite. For instance, at a heating rate of 5 °C·min^−1^, the estimated values of $E_{A}$ for the pure DSP-5, 50%DSP, 25%DSP-5, and pure HDPE samples range from 40.419 to 91.080 kJ·mol^−1^, as determined by using the BD kinetic model in conjunction with the D2 diffusion model. Table 3 further indicates that the pre-exponential factor (A) increases significantly with the rise in activation energy ($E_{A}$), suggesting a rapid reaction achieved at elevated temperatures for samples with a higher HDPE proportion. Additionally, the estimated values for the changes in enthalpy (∆H) and Gibbs free energy (∆G) were consistently positive, while negative values were estimated for the change in entropy (∆S) throughout the analysis using the Borchardt and Daniels kinetic model.

**Figure 6:**
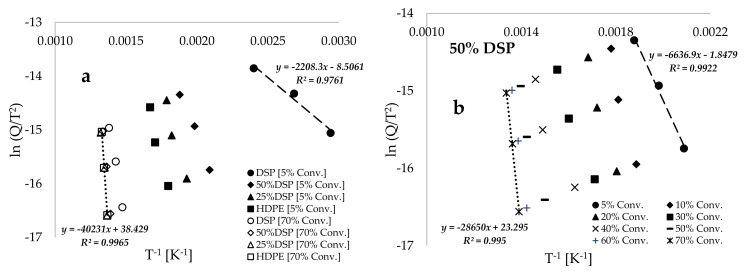

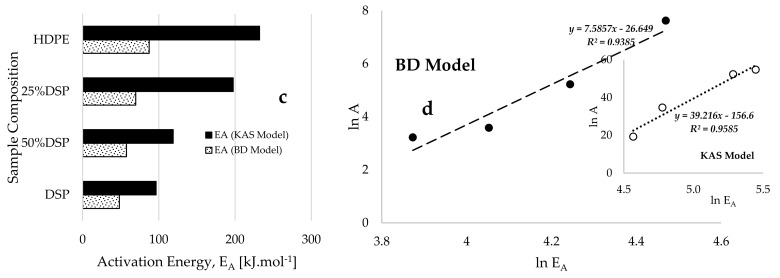
Plots of KAS’ ln (Q/T2) against the inverse of conversion temperature (T^−1^ [K^−1^]) for (**a**) varied sample compositions at 5 wt% and 70 wt%, (**b**) a 50%DSP sample at various conversions ranging from 5 wt% to 70 wt%, (**c**) estimated overall activation energy values plotted against different sample compositions, and (**d**) the kinetic composition effect illustrated by plots of ln*A* against ln*E_A_* for both the BD and KAS kinetic models.

Figure 6a presents plots of KAS’ ln(Q/T^2^) against the inverse of conversion temperature (T^−1^, [K^−1^]) for samples of varied compositions at both the lowest (5 wt%) and highest (70 wt%) conversions selected. This exhibits an increasing linear inverse trend towards temperature minima with an increase in DSP proportion within the blend, as well as a reduction in sample mass conversion. Figure 6b provides a more comprehensive view of the observations in Figure 6a, revealing the consistent shift to higher temperatures with the increase in mass profile conversion during the thermal decomposition of the 50%DSP sample. Notably, the slope of the trend at 5% conversion differs from those observed for 10%, 20%, and 30% conversions, suggesting that at lower conversion levels, the material exhibits more amorphous characteristics at this low temperature, which subsequently transitions to a semi-crystalline and eventually fully crystalline state at higher temperatures [23]. According to reported studies in [49,51], the dehydration stage of material decomposition is characterized by reversibility and may also be referred to as a second-order transition, while the combustion stage is distinguished by a significant loss in material content, categorizing it as a first-order and irreversible transition.

Figure 6c and Table 4 present the estimated average values of activation energy ($E_{A}$) obtained for this process by using the isothermal KAS model-free kinetic method. These values are 96.316, 118.804, 197.336, and 232.034 kJ.mol^−1^ for the pure DSP, 50%DSP, 25%DSP and pure HDPE samples, respectively, which are higher than those estimated via the BD model-fitting method. These variations in the estimated kinetic data may be attributed to the non-linear deviations observed in the linear–inverse trends associated with the application of the BD model-fitting methods at lower conversion levels [52]. This methodology resulted in reduced coefficients of determination when a linear model was employed, in contrast to the significantly better fits achieved through the application of the KAS model-fitting methods. Table 4 indicates that the pre-exponential factor (A) and the estimated values of thermodynamic parameters increase with the increase in the percentage conversion and the HDPE plastic proportion in the sample. Figure 6d illustrates the kinetic compensation effect, establishing the relationship between the natural logarithm of the pre-exponential factor (A) and the natural logarithm of the activation energy ($E_{A}$). This approach is derived from a study reported by Janković et al. [53], which demonstrated a linear relationship between the pre-exponential factor and activation energy ($lnA=\alpha +\beta .lnE_{A}$). The data presented in Figure 6d suggest that the application of the proposed model in [53] to the estimated data in this study indicates a linear relation between the pre-exponential factor and activation energy, as estimated by using both the BD model-fitting and KAS model-free kinetic models. With reasonable values of the coefficient of determination estimated for the two models, the plots in Figure 6d suggest that both pyrolysis and co-pyrolysis processes facilitate the direct rapid conversion and disintegration of the samples from lower to higher degradation temperatures [23,48], despite the study being conducted at the low heating rates of 2, 5, and 10 °C.min^−1^. Finally, the use of both the BD model-fitting and KAS model-free kinetic methods resulted in the estimation of positive values for changes in enthalpy (∆H) and Gibbs free energy (∆G) throughout the analysis, suggesting that the pyrolysis and co-pyrolysis processes are endothermic and non-spontaneous, respectively. In contrast, the estimated values of changes in entropy (∆S) derived from the two selected kinetic models remained negative and approached zero, suggesting that the process is stable or exhibits a lower degree of disorder.

While the current study provided kinetic and thermodynamic data on the pyrolysis and co-pyrolysis of DSP, HDPE, and their blends at the low heating rates of 2, 5, and 10 °C·min^−1^, a previously published report referenced as [15] presented thermograms, kinetics, and thermodynamic data for the thermal degradation of similar materials at higher heating rates, 10, 20, and 40 °C·min^−1^. The study [15] revealed that a total degradation time of 2925 s was recorded for these materials at a maximum degradation temperature of 600 °C, specifically at a heating rate of 20 °C·min^−1^. Furthermore, it noted a 17 wt% loss for the thermal degradation of the pure DSP biomass, occurring within a temperature range of 35 to 285 °C, while a 1.0 wt% loss was observed for HDPE degradation at 355 °C, and a 5 wt% loss was recorded for the 50%DSP composite sample at 285 °C. Additionally, the overall activation energy estimated for the process ranged between 22.903 and 101.515 kJ·mol⁻^1^, determined by using the Coats–Redfern model-fitting kinetic method. The current study indicated a degradation time of 6900 s for the thermal decomposition of these materials at a heating rate of 5 °C·min^−1^, resulting in a 15 wt% loss for the thermal degradation of the DSP biomass material with a degradation temperature between 25 and 235 °C. In this slow pyrolysis process, the propagation stage of HDPE degradation commenced at a higher temperature of 280 °C (resulting in a 1.0 wt% loss), whereas 50%DSP composite degradation began at 245 °C (resulting in a 5 wt% loss). Thus, the data obtained for these two pyrolysis processes at varying heating rates suggest that the slow pyrolysis conducted in this study resulted in a significantly longer degradation time due to the dominant effects of pyrolysis temperature on the materials [54,55]. Cetin et al. [56] noted that fast pyrolysis often leads to considerable loss of the material’s structural complexity due to the local melting of structures and rapid phase transformation, while slow pyrolysis favors the retention of structural rigidity. In terms of thermal stability, slight variations in the thermograms exhibited a shift to lower temperatures for the slow pyrolysis processes (low heating rates), accompanied by slight differences in the estimated values of kinetic and thermodynamic parameters. A research study by Crombie et al. [54] indicated that there was no notable impact on the concentration of stable carbon in biochar obtained from the pyrolysis of rice, pine, and wheat straw when conducted at heating rates ranging from 1 to 100 °C·min^−1^. Therefore, it is recommended to conduct pyrolysis and co-pyrolysis processes for these materials (DSP, HDPE, and their blends) at significantly higher heating rates, such as 50, 100, and 150 °C·min⁻^1^, as this may result in wider deviations in kinetic and thermodynamic parameters compared with the slow pyrolysis processes conducted in this study.

## 6. Conclusions

This study presents an analysis of the thermal decomposition of date seed powder (DSP), high-density polyethylene (HDPE), and their composites utilizing thermogravimetric analysis (TGA) data collected at degradation temperatures ranging from 25 to 600 °C and at low heating rates of 2, 5, and 10 °C·min^−1^. Fourier transform infrared (FTIR) spectroscopy conducted on the samples revealed that the DSP biomass is predominantly characterized by hemicellulose, cellulose, and lignin contents, while the HDPE plastic exhibited C-H stretching vibrations indicative of alkanes and various other hydrocarbons. The findings of the study indicate that the presence of hemicellulose and cellulose in the DSP biomass facilitates a synergistic interaction between the materials during co-pyrolysis, thereby diminishing the thermal stability of the HDPE plastic. Furthermore, the presence of lignin in the DSP biomass was observed to impede the degradation of the composite samples at elevated temperatures.

The application of machine learning through convolutional deep neural networks (CDNN) reliably provided learning algorithms and predictions that closely aligned with experimental measurements ($R^{2}\sim 0.939$), utilizing 27,456 epochs and varying learning rates between 5 and 2. This resulted in an optimized CDNN framework characterized by two hidden layers, consisting of three neurons. The modeling technique yielded a mean absolute error (MAE) of 4.96 and a mean bias error (MBE) of 0.165. Following a sensitivity analysis, degradation temperature was identified as the most significant parameter influencing the process, succeeded by sample composition, degradation, and heating rate in descending order of importance. A comparative analysis between the trained machine learning convolutional deep neural network (CDNN) model and a formulated model utilizing the multiple linear regression model (MLRM) indicates that the CDNN model provides more favorable predictions. The results suggest a significantly improved representation of the non-linear trends observed at both the initiation and termination stages of the degradation processes. A kinetic analysis of the process identified D2 and D3 diffusion models as possible solid-state reaction mechanisms. The estimated values for activation energy ranged from 40.419 to 91.080 kJ.mol^−1^ using the BD model-fitting method and from 96.316 to 226.286 kJ.mol^−1^ using the KAS model-free kinetic methods. Additionally, kinetic compensation effects indicated a rapid increase in the pre-exponential factor with the increase in activation energy, while the thermodynamic parameters estimated throughout the study indicated that the process is endothermic, non-spontaneous, and stable. This clearly suggests that the incorporation of DSP into HDPE enhances its degradation, potentially facilitating more sustainable and cost-effective pyrolysis.

The trained CDNN model in this study has the potential to provide valuable information to environmental engineers and plastic manufacturers in facilitating the optimal design of bio-based plastics (DSP/HDPE) with enhanced thermal properties. Nevertheless, it is essential to assess additional physical and chemical characteristics of the composites, such as tensile strength, durability, reactivity, chemical stability, melting point, and conductivity. This comprehensive evaluation will assist in selecting suitable material compositions that can enhance the efficacy of DSP as a biocatalyst accelerator for HDPE polymer degradation.

## Figures and Tables

**Table 1:** Estimated synaptic weights and biases, along with a statistical comparison of modeling and experimental data.

**b_1_**	**w_1_**	**w_2_**	**w_3_**	**w_4_**		**EXPT**	**CDNN**
−5.738	−0.923	7.205	0.262	0.829	Mean value	68.282	68.143
						1.418	1.365
**b_2_**	**w_5_**	**w_6_**	**w_7_**	**w_8_**	Mean absolute error (MAE) or L1 loss	4.906	
−5.738	−0.923	7.205	0.262	0.829	Mean bias error (MBE)	0.165	
							
**b_6_**	**w_21_**	**w_22_**	The remaining synaptic weights and biases in Equations (1.1) to (2.3) of the Supplementary Materials are zero.		Final true error	4.882%	
−0.247	−11.628	−11.628			Learning rate	5 → 2	
					Total training time	5 days	
**b_11_**	**w_46_**				Initial loss function	44.089	
−1.606	14.390				Final loss function	2.153	

**Table 2:** Selected solid-state reaction mechanisms (SSRMs).

Reaction Mechanisms	$g\left(x_{i}\right)$	$f\left(x_{i}\right)$
First-order reaction model [F1]	$-ln\left(1-x_{i}\right)$	$\left(1-x_{i}\right)$
Second-order reaction model [F2]	${{(1-x}_{i})}^{-1}-1$	${{(1-x}_{i})}^{2}$
Third-order reaction model [F3]	$\left[{{(1-x}_{i})}^{-2}-1\right]/2$	${{(1-x}_{i})}^{3}$
Geometrical contraction model (contracting sphere [R2])	${{1-(1-x}_{i})}^{1/2}$	${{2(1-x}_{i})}^{1/2}$
Geometrical contraction model (contracting cylinder [R3])	${{1-(1-x}_{i})}^{1/3}$	${{3(1-x}_{i})}^{2/3}$
Avarami–Erofeev (A2)	${{[-ln(1-x}_{i})]}^{1/2}$	${{2\left(1-x_{i}\right)[-ln(1-x}_{i})]}^{1/2}$
Avarami–Erofeev (A3)	$[{{-ln(1-x}_{i})]}^{1/3}$	${{3\left(1-x_{i}\right)[-ln(1-x}_{i})]}^{2/3}$
Avarami–Erofeev (A4)	$[{{-ln(1-x}_{i})]}^{1/4}$	${{4\left(1-x_{i}\right)[-ln(1-x}_{i})]}^{3/4}$
Two-dimension diffusion model (D2)	$[\left(1-x_{i}\right)ln\left(1-x_{i}\right)]+x_{i}$	${{[-ln(1-x}_{i})]}^{-1}$
Three-dimension diffusion model (Jander [D3])	${\left[{{1-(1-x}_{i})}^{1/3}\right]}^{2}$	$3{{(1-x}_{i})}^{2/3}/[2\left({{1-(1-x}_{i})}^{1/3}\right)]$

**Table 3:** Estimated kinetic and thermodynamic parameters using Borchardt and Daniels’ model-fitting method for constant heating rates.

Sample	SSRM	R^2^	E_A_	A	ΔS	ΔH	ΔG	Sample	SSRM	R^2^	E_A_	A	ΔS	ΔH	ΔG
**DSP-2**	**F1**	0.844	26.181	1.187	−0.285	20.157	226.587	**HDPE-2**	**F1**	0.911	41.787	4.008	−0.275	35.710	236.623
	**F2**	0.930	42.853	66.138	−0.251	36.829	219.043		**F2**	0.878	53.326	42.536	−0.255	47.249	233.808
	**F3**	0.952	63.850	10,040.122	−0.210	57.826	209.784		**F3**	0.844	66.714	650.307	−0.233	60.637	230.623
	**R2**	0.763	19.837	0.126	−0.304	13.813	233.758		**R2**	0.925	36.750	0.711	−0.289	30.673	242.094
	**R3**	0.703	21.803	0.136	−0.303	15.779	235.254		**R3**	0.921	38.375	0.663	−0.290	32.298	244.149
	**A2**	0.705	8.382	0.035	−0.314	2.358	230.059		**A2**	0.856	15.885	0.060	−0.310	9.808	236.284
	**A3**	0.330	2.450	0.011	−0.324	−3.574	231.218		**A3**	0.737	7.251	0.015	−0.321	1.174	236.172
	**D2**	0.800	45.022	11.509	−0.266	38.998	231.746		**D2**	0.945	80.191	573.550	−0.234	74.114	244.863
	**D3**	0.845	53.023	18.195	−0.262	46.999	236.987		**D3**	0.937	86.765	493.560	−0.235	80.688	252.350
**50% DSP-2**	**F1**	0.933	27.267	0.623	−0.290	21.286	230.072	**25% DSP-2**	**F1**	0.891	31.160	0.679	−0.290	25.068	237.320
	**F2**	0.962	37.963	7.092	−0.270	31.981	226.223		**F2**	0.851	40.444	4.966	−0.273	34.352	234.482
	**F3**	0.964	50.698	125.769	−0.246	44.716	221.759		**F3**	0.813	51.235	49.533	−0.254	45.143	231.259
	**R2**	0.902	22.749	0.112	−0.304	16.767	235.836		**R2**	0.909	27.118	0.142	−0.303	21.025	242.811
	**R3**	0.914	24.194	0.103	−0.305	18.212	237.780		**R3**	0.903	28.421	0.125	−0.304	22.328	244.870
	**A2**	0.849	8.643	0.024	−0.317	2.662	231.024		**A2**	0.791	10.580	0.025	−0.317	4.487	236.951
	**A3**	0.501	2.435	0.008	−0.326	−3.547	231.341		**A3**	0.514	3.719	0.008	−0.326	−2.374	236.828
	**D2**	0.922	52.506	13.820	−0.264	46.525	236.776		**D2**	0.939	61.566	25.420	−0.260	55.474	245.655
	**D3**	0.939	58.368	11.797	−0.266	52.387	243.585		**D3**	0.928	66.843	17.641	−0.263	60.750	253.157
**DSP-5**	**F1**	0.823	18.537	0.535	−0.291	12.898	210.256	**HDPE-5**	**F1**	0.927	48.887	28.220	−0.259	42.703	235.198
	**F2**	0.878	29.341	3.061	−0.276	23.702	211.225		**F2**	0.892	60.962	130.836	−0.246	54.777	237.786
	**F3**	0.860	39.441	10,040.122	−0.209	33.802	175.675		**F3**	0.861	76.475	2751.894	−0.221	70.290	234.461
	**R2**	0.857	17.428	0.069	−0.308	11.789	220.706		**R2**	0.932	41.794	1.485	−0.283	35.609	246.315
	**R3**	0.865	18.544	0.062	−0.309	12.905	222.453		**R3**	0.929	43.669	1.436	−0.284	37.485	248.397
	**A2**	0.730	6.535	0.022	−0.318	0.896	216.320		**A2**	0.869	18.137	0.083	−0.307	11.953	240.486
	**A3**	0.268	1.736	0.009	−0.325	−3.903	216.721		**A3**	0.754	8.312	0.017	−0.320	2.128	240.407
	**D2**	0.898	40.419	3.799	−0.275	34.780	221.087		**D2**	0.950	91.080	2800.756	−0.221	84.895	248.958
	**D3**	0.902	44.950	1296.787	−0.226	39.311	192.725		**D3**	0.944	98.679	2805.521	−0.221	92.494	256.546
**50% DSP-5**	**F1**	0.958	31.350	2.346	−0.279	25.248	230.257	**25% DSP-5**	**F1**	0.939	43.095	11.673	−0.266	36.936	234.060
	**F2**	0.955	41.732	10.015	−0.267	35.631	231.785		**F2**	0.884	52.570	35.458	−0.257	46.411	236.692
	**F3**	0.932	54.999	174.033	−0.244	48.897	227.631		**F3**	0.846	66.696	608.225	−0.233	60.537	233.312
	**R2**	0.951	25.768	0.157	−0.302	19.667	241.190		**R2**	0.943	35.171	0.523	−0.292	29.012	245.260
	**R3**	0.956	27.294	0.146	−0.302	21.193	243.154		**R3**	0.937	36.868	0.493	−0.292	30.709	247.327
	**A2**	0.908	9.859	0.027	−0.316	3.757	235.937		**A2**	0.838	13.877	0.042	−0.313	7.718	239.489
	**A3**	0.611	2.968	0.009	−0.326	−3.134	236.122		**A3**	0.576	5.022	0.010	−0.325	−1.137	239.430
	**D2**	0.965	59.216	29.384	−0.258	53.114	242.701		**D2**	0.966	79.548	465.189	−0.235	73.388	247.815
	**D3**	0.970	65.403	25.225	−0.260	59.301	249.819		**D3**	0.956	86.424	418.570	−0.236	80.265	255.342
**DSP-10**	**F1**	0.808	32.898	267.962	−0.240	27.073	194.933	**HDPE-10**	**F1**	0.936	49.021	59.415	−0.253	42.769	232.786
	**F2**	0.878	42.812	30.516	−0.258	36.987	217.503		**F2**	0.937	62.651	181.498	−0.243	56.399	239.435
	**F3**	0.925	57.967	979.894	−0.229	52.142	212.448		**F3**	0.922	79.565	4921.874	−0.216	73.313	235.715
	**R2**	0.732	24.810	0.239	−0.298	18.984	227.756		**R2**	0.942	41.932	1.554	−0.283	35.680	248.477
	**R3**	0.754	26.511	0.236	−0.298	20.686	229.513		**R3**	0.943	43.944	1.541	−0.283	37.692	250.543
	**A2**	0.576	8.702	0.030	−0.315	2.877	223.719		**A2**	0.916	18.633	0.092	−0.307	12.381	242.877
	**A3**	0.093	1.557	0.008	−0.326	−4.268	224.346		**A3**	0.837	8.782	0.019	−0.320	2.530	242.938
	**D2**	0.789	58.851	91.968	−0.248	53.025	227.115		**D2**	0.946	90.656	2716.622	−0.221	84.404	250.522
	**D3**	0.819	65.755	101.793	−0.248	59.929	233.428		**D3**	0.949	98.812	3013.762	−0.220	92.560	258.029
**50% DSP-10**	**F1**	0.965	32.385	4.818	−0.274	26.164	230.828	**25% DSP-10**	**F1**	0.958	35.545	6.279	−0.271	29.251	234.734
	**F2**	0.957	42.751	10.238	−0.267	36.530	236.505		**F2**	0.909	45.710	10.849	−0.267	39.416	241.458
	**F3**	0.930	56.211	174.993	−0.244	49.990	232.307		**F3**	0.874	58.403	145.801	−0.245	52.108	237.797
	**R2**	0.963	26.539	0.163	−0.302	20.318	246.050		**R2**	0.959	30.163	0.220	−0.299	23.869	250.454
	**R3**	0.967	28.091	0.151	−0.302	21.870	248.059		**R3**	0.954	31.673	0.200	−0.300	25.379	252.544
	**A2**	0.924	10.179	0.028	−0.316	3.958	240.680		**A2**	0.866	12.061	0.031	−0.316	5.767	244.712
	**A3**	0.641	3.111	0.009	−0.326	−3.110	240.858		**A3**	0.617	4.463	0.009	−0.326	−1.831	244.728
	**D2**	0.974	60.915	31.761	−0.258	54.694	247.627		**D2**	0.978	67.962	62.112	−0.252	61.668	252.727
	**D3**	0.977	67.203	27.106	−0.259	60.982	254.901		**D3**	0.971	74.079	49.060	−0.254	67.784	260.328

N.B.: E_A_ is the activation energy [kJ.mol^−1^]; A is the pre-exponential factor [ min^−1^]; ΔS is the change in entropy [kJ.mol^−1^.K^−1^]; ΔH is the change in enthalpy [kJ.mol^−1^]; ΔG is the change in Gibbs free energy [kJ.mol^−1^].

**Table 4:** Estimated kinetic and thermodynamic parameters using the Kissinger–Akahira–Sunose model-free method for variable heating rates.

Sample	% Conv.	R^2^	E_A_	A	ΔS	ΔH	ΔG	Sample	% Conv.	R^2^	E_A_	A	ΔS	ΔH	ΔG
**DSP**	5	0.976	18.360	0.046	−0.307	14.895	143.004	**HDPE**	5	0.965	90.423	2.672 × 10^4^	−0.200	85.443	205.251
	10	0.992	77.120	1.196 × 10^5^	−0.186	72.765	170.420		10	0.958	135.527	5.869 × 10^8^	−0.117	130.397	202.667
	20	0.992	106.452	1.025 × 10^8^	−0.131	101.809	174.873		20	0.990	180.472	4.533 × 10^12^	−0.043	175.114	202.872
	30	0.948	101.738	5.156 × 10^7^	−0.137	97.017	174.625		30	0.946	223.705	1.176 × 10^15^	0.002	217.851	216.157
	40	0.995	112.730	6.913 × 10^8^	−0.115	107.889	175.014		40	0.987	256.961	1.423 × 10^17^	0.042	250.894	220.255
	50	0.912	103.035	4.686 × 10^7^	−0.138	97.942	182.547		50	0.993	272.982	1.650 − 10^18^	0.062	266.818	220.687
	60	0.992	120.744	5.109 × 10^8^	−0.119	115.304	193.031		60	0.986	361.726	4.558 × 10^24^	0.186	355.545	217.645
	70	0.992	130.347	3.868 × 10^8^	−0.122	124.323	212.673		70	0.997	334.481	4.050 − 10^22^	0.146	328.228	218.325
**Average**		**0.975**	**96.316**	**2.238 × 10^8^**	**−0.157**	**91.493**	**178.273**	**Average**		**0.978**	**232.034**	**5.748 × 10^23^**	**0.010**	**226.286**	**212.982**
**50%DSP**	5	0.992	55.179	92.670	−0.246	50.752	181.816	**25% DSP**	5	0.952	55.183	7.335 × 10^3^	−0.210	50.528	168.233
	10	0.970	111.183	4.973 × 10^7^	−0.137	106.511	183.428		10	0.994	123.521	2.019 × 10^8^	−0.126	118.615	192.756
	20	0.979	98.155	3.277 × 10^6^	−0.160	93.215	188.264		20	0.967	128.958	3.065 × 10^8^	−0.123	123.674	201.717
	30	0.976	71.781	7.466 × 10^3^	−0.211	66.426	202.467		30	0.963	172.582	2.612 × 10^11^	−0.067	166.829	213.460
	40	0.905	62.279	8.139 × 10^2^	−0.230	56.584	214.255		40	0.994	235.461	1.065 × 10^16^	0.021	229.541	214.850
	50	0.955	112.555	6.948 × 10^6^	−0.155	106.605	217.703		50	0.998	236.833	5.317 × 10^15^	0.015	230.707	219.968
	60	0.999	201.099	2.403 × 10^13^	−0.030	194.987	217.262		60	0.931	347.326	4.213 × 10^23^	0.166	341.124	217.534
	70	0.995	238.196	9.201 x10^15^	0.019	231.975	217.754		70	0.926	278.827	1.623 × 10^16^	0.024	272.532	254.643
**Average**		**0.971**	**118.804**	**1.153 × 10^15^**	**−0.144**	**113.382**	**202.869**	**Average**		**0.966**	**197.336**	**5.267 × 10^22^**	**−0.038**	**191.694**	**210.395**

N.B.: E_A_ is the activation energy [kJ.mol^−1^]; A is the pre-exponential factor [ min^−1^]; ΔS is the change in entropy [kJ.mol^−1^.K^−1^]; ΔH is the change in enthalpy [kJ.mol^−1^]; ΔG is the change in Gibbs free energy [kJ.mol^−1^].

## Data Availability

Data are included within the article.

## Supplementary Materials

The following supporting information can be downloaded at: https://www.mdpi.com/article/10.3390/polym17060740/s1, Figure S1. A DNN framework showing typical inputs, hidden layers and an output neuron.
