# Supplementary material for: Low-Heating-Rate Thermal Degradation of Date Seed Powder and HDPE Plastic: Machine Learning CDNN, MLRM, and Thermokinetic Analysis
Source: Polymers (Basel). 2025 Mar 11;17(6):740. doi: 10.3390/polym17060740 (PMC11945590; doi:10.3390/polym17060740)
Supplement: Supplementary file 1 [file polymers-17-00740-s001.zip › polymers-3492476-supplementary.pdf]

Low-Heating-Rate Thermal Degradation of Date Seed Powder and HDPE Plastic: Machine Learning CDNN, MLRM, and Thermokinetic Analysis

Zaid Abdulhamid Alhulaybi Albin Zaid * and Abdulrazak Jinadu Otaru *

Department of Chemical Engineering, College of Engineering, King Faisal University, Al Ahsa 31982, Saudi Arabia
* Correspondence: zalhulaybi@kfu.edu.sa (Z.A.A.A.Z.); aotaru@kfu.edu.sa (A.J.O.)

Feedforward and Backpropagation Algorithms for a Deep Neural Network (DNN)

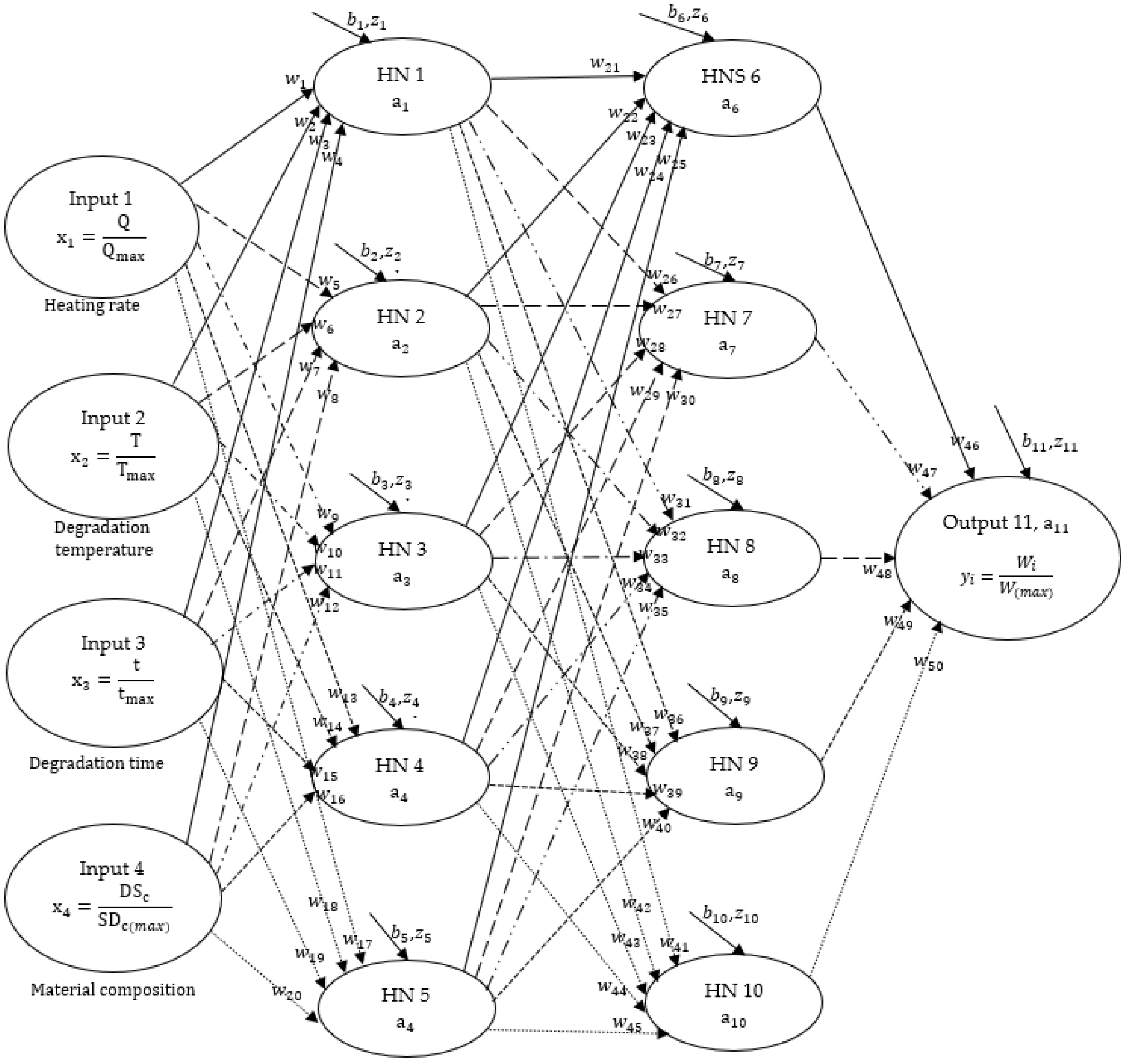

Figure S1. A DNN framework showing typical inputs, hidden layers and an output neuron.

Applying the general artificial neural networks (ANN) Equations (S1–S3) in the main article to the framework in Figure S1 results in the following equations:

$z_{11} = b_{11} + w_{46}.a_6 + w_{47}.a_7 + w_{48}.a_8 + w_{49}.a_9 + w_{50}.a_{10}$ Equation S1.0

$a_{11} = \sigma'(z_{11}) = \frac{1}{(1+e^{-z_{11}})}$ Equation S1.2

$C_i = (y - a_{11})^2$ Equation S1.3

where $a_6 = \sigma'(z_6) = \frac{1}{(1+e^{-z_6})}$

& $z_6 = b_6 + w_{21}.a_1 + w_{22}.a_2 + w_{23}.a_3 + w_{24}.a_4 + w_{25}.a_5$ Equation S1.4

$a_7 = \sigma'(z_7) = \frac{1}{(1+e^{-z_7})}$ & $z_7 = b_7 + w_{26}.a_1 + w_{27}.a_2 + w_{28}.a_3 + w_{29}.a_4 + w_{30}.a_5$ Equation S1.5

$a_8 = \sigma'(z_8) = \frac{1}{(1+e^{-z_8})}$ & $z_8 = b_8 + w_{31}.a_1 + w_{32}.a_2 + w_{33}.a_3 + w_{34}.a_4 + w_{35}.a_5$ Equation S1.6

$a_9 = \sigma'(z_9) = \frac{1}{(1+e^{-z_9})}$ & $z_9 = b_9 + w_{36}a_1 + w_{37}a_2 + w_{38}a_3 + w_{39}a_4 + w_{40}a_5$ Equation S1.7

$a_{10} = \sigma'(z_{10}) = \frac{1}{(1+e^{-z_{10}})}$ & $z_{10} = b_{10} + w_{41}a_1 + w_{42}a_2 + w_{43}a_3 + w_{44}a_4 + w_{45}a_5$ Equation S1.8

$a_1 = \sigma'(z_1) = \frac{1}{(1+e^{-z_1})}$ & $z_1 = b_1 + w_1.x_1 + w_2.x_2 + w_3.x_3 + w_4.x_4$ Equation S1.9

$a_2 = \sigma'(z_2) = \frac{1}{(1+e^{-z_2})}$ & $z_2 = b_2 + w_5.x_1 + w_6.x_2 + w_7.x_3 + w_8.x_4$ Equation S2.0

$a_3 = \sigma'(z_3) = \frac{1}{(1+e^{-z_3})}$ & $z_3 = b_3 + w_9.x_1 + w_{10}.x_2 + w_{11}.x_3 + w_{12}.x_4$ Equation S2.1

$a_4 = \sigma'(z_4) = \frac{1}{(1+e^{-z_4})}$ & $z_4 = b_4 + w_{13}.x_1 + w_{14}.x_2 + w_{15}.x_3 + w_{16}.x_4$ Equation S2.2

$a_5 = \sigma'(z_5) = \frac{1}{(1+e^{-z_5})}$ & $z_5 = b_5 + w_{17}.x_1 + w_{18}.x_2 + w_{19}.x_3 + w_{20}.x_4$ Equation S2.3

Loss Function for the DNN Framework in Figure S1

The following steps are thus followed for the formulation of the learning algorithms for the loss function or cost optimisation function.

1. **The overall cost function on activation function 11**

The estimation of the cost function at this stage is dependent on the knowledge of the changes in bias (Δb) and weights (Δw) and these can be formulated as a test function of the input activation functions ($a_6 - a_{10}$), learning rate (L), sum weights and real output data.

$\Delta b = -L.\frac{\partial C}{\partial b}$ & $\Delta w = -L.\frac{\partial C}{\partial w}$ Equation S3.0

where $\frac{\partial C}{\partial b} = \left(\frac{\partial C}{\partial a}\right)\left(\frac{\partial a}{\partial z}\right)\left(\frac{\partial z}{\partial b}\right)$ & $\frac{\partial C}{\partial w} = \left(\frac{\partial C}{\partial a}\right)\left(\frac{\partial a}{\partial z}\right)\left(\frac{\partial z}{\partial w}\right)$ Equation S3.1

$\left(\frac{\partial C}{\partial a_{11}}\right) = 2(a_{11} - y)$; $\left(\frac{\partial a_{11}}{\partial z_{11}}\right) = \sigma'(z_{11}) = \frac{e^{z_{11}}}{(1+e^{z_{11}})^2}$; $\left(\frac{\partial z_{11}}{\partial w_{51}}\right) = a_6$, $\left(\frac{\partial z_{11}}{\partial w_{52}}\right) = a_7$,

$\left(\frac{\partial z_{11}}{\partial w_{53}}\right) = a_8$, $\left(\frac{\partial z_{11}}{\partial w_{54}}\right) = a_9$, $\left(\frac{\partial z_{11}}{\partial w_{55}}\right) = a_{10}$ & $\left(\frac{\partial z_{11}}{\partial b_{11}}\right) = 1$ Equation S3.2

$$\left(\frac{\partial C}{\partial b_{11}}\right) = \left(\frac{\partial C}{\partial a_{11}}\right)\left(\frac{\partial a_{11}}{\partial z_{11}}\right)\left(\frac{\partial z_{11}}{\partial b_{11}}\right) \quad \text{Equation S3.3a}$$

$$\left(\frac{\partial C}{\partial w_{46}}\right) = \left(\frac{\partial C}{\partial a_{11}}\right)\left(\frac{\partial a_{11}}{\partial z_{11}}\right)\left(\frac{\partial z_{11}}{\partial w_{46}}\right) \quad \text{Equation S3.3b}$$

$$\left(\frac{\partial C}{\partial w_{47}}\right) = \left(\frac{\partial C}{\partial a_{11}}\right)\left(\frac{\partial a_{11}}{\partial z_{11}}\right)\left(\frac{\partial z_{11}}{\partial w_{47}}\right) \quad \text{Equation S3.3c}$$

$$\left(\frac{\partial C}{\partial w_{48}}\right) = \left(\frac{\partial C}{\partial a_{11}}\right)\left(\frac{\partial a_{11}}{\partial z_{11}}\right)\left(\frac{\partial z_{11}}{\partial w_{48}}\right) \quad \text{Equation S3.3d}$$

$$\left(\frac{\partial C}{\partial w_{49}}\right) = \left(\frac{\partial C}{\partial a_{11}}\right)\left(\frac{\partial a_{11}}{\partial z_{11}}\right)\left(\frac{\partial z_{11}}{\partial w_{49}}\right) \quad \text{Equation S3.3e}$$

$$\left(\frac{\partial C}{\partial w_{50}}\right) = \left(\frac{\partial C}{\partial a_{11}}\right)\left(\frac{\partial a_{11}}{\partial z_{11}}\right)\left(\frac{\partial z_{11}}{\partial w_{50}}\right) \quad \text{Equation S3.3f}$$

Hence: $\Delta b_{11} = -L\left(\sum_{i=1}^{n}\left(\frac{\partial C}{\partial b_{11}}\right)\right)/n \;\;\&\;\; b_{11} = b_{11} + \Delta b_{11}$ Equation S3.4a

$$\Delta w_{46} = -L\left(\sum_{i=1}^{n}\left(\frac{\partial C}{\partial w_{46}}\right)\right)/n \;\;\&\; w_{46(New)} = w_{46(Old)} + \Delta w_{46} \quad \text{Equation S3.4b}$$

$$\Delta w_{47} = -L\left(\sum_{i=1}^{n}\left(\frac{\partial C}{\partial w_{47}}\right)\right)/n \;\;\&\; w_{47(New)} = w_{47(Old)} + \Delta w_{47} \quad \text{Equation S3.4c}$$

$$\Delta w_{48} = -L\left(\sum_{i=1}^{n}\left(\frac{\partial C}{\partial w_{48}}\right)\right)/n \;\;\&\; w_{48(New)} = w_{48(Old)} + \Delta w_{48} \quad \text{Equation S3.4d}$$

$$\Delta w_{49} = -L\left(\sum_{i=1}^{n}\left(\frac{\partial C}{\partial w_{49}}\right)\right)/n \;\;\&\; w_{49(New)} = w_{49(Old)} + \Delta w_{49} \quad \text{Equation S3.4e}$$

$$\Delta w_{50} = -L\left(\sum_{i=1}^{n}\left(\frac{\partial C}{\partial w_{50}}\right)\right)/n \;\;\&\; w_{50(New)} = w_{50(Old)} + \Delta w_{50} \quad \text{Equation S3.4f}$$

2. The overall cost function on hidden neurons (HNL) $a_6 - a_{10}$

The changes in bias and weights at this stage is done as a test function of activation with respect to sum weight and sum weight with respect to individual weights and biases.

$$\frac{\partial C}{\partial b} = \left(\frac{\partial C}{\partial a}\right)\left(\frac{\partial a}{\partial z}\right)\left(\frac{\partial z}{\partial b}\right) \;\&\; \frac{\partial C}{\partial w} = \left(\frac{\partial C}{\partial a}\right)\left(\frac{\partial a}{\partial z}\right)\left(\frac{\partial z}{\partial w}\right) \quad \text{Equation S4.1}$$

where $\left(\frac{\partial z_{11}}{\partial a_6}\right) = w_{46}$; $\left(\frac{\partial z_{11}}{\partial a_7}\right) = w_{47}$; $\left(\frac{\partial z_{11}}{\partial a_8}\right) = w_{48}$; $\left(\frac{\partial z_{11}}{\partial a_9}\right) = w_{49}$; $\left(\frac{\partial z_{11}}{\partial a_{10}}\right) = w_{50}$ Equation S4.2

$$\left(\frac{\partial C}{\partial a_6}\right) = \left(\frac{\partial C}{\partial a_{11}}\right)\left(\frac{\partial a_{11}}{\partial z_{11}}\right)\left(\frac{\partial z_{11}}{\partial a_6}\right) \quad \text{where} \quad \left(\frac{\partial a_{11}}{\partial z_{11}}\right) = \sigma'(z_{11}) = \frac{e^{z_{11}}}{(1+e^{z_{11}})^2} \quad \text{Equation S4.3a}$$

$$\left(\frac{\partial C}{\partial a_7}\right) = \left(\frac{\partial C}{\partial a_{11}}\right)\left(\frac{\partial a_{11}}{\partial z_{11}}\right)\left(\frac{\partial z_{11}}{\partial a_7}\right) \quad \text{Equation S4.3b}$$

$$\left(\frac{\partial C}{\partial a_8}\right) = \left(\frac{\partial C}{\partial a_{11}}\right)\left(\frac{\partial a_{11}}{\partial z_{11}}\right)\left(\frac{\partial z_{11}}{\partial a_8}\right) \quad \text{Equation S4.3c}$$

$$\left(\frac{\partial C}{\partial a_9}\right) = \left(\frac{\partial C}{\partial a_{11}}\right)\left(\frac{\partial a_{11}}{\partial z_{11}}\right)\left(\frac{\partial z_{11}}{\partial a_9}\right) \quad \text{Equation S4.3d}$$

$$\left(\frac{\partial C}{\partial a_{10}}\right) = \left(\frac{\partial C}{\partial a_{11}}\right)\left(\frac{\partial a_{11}}{\partial z_{11}}\right)\left(\frac{\partial z_{11}}{\partial a_{10}}\right) \quad \text{Equation S4.3e}$$

$$\left(\frac{\partial C}{\partial b_6}\right) = \left(\frac{\partial C}{\partial a_6}\right)\left(\frac{\partial a_6}{\partial z_6}\right)\left(\frac{\partial z_6}{\partial b_6}\right) \quad \text{where} \left(\frac{\partial z_6}{\partial b_6}\right) = 1 \quad \text{Equation S4.4a}$$

$$\left(\frac{\partial C}{\partial w_{21}}\right) = \left(\frac{\partial C}{\partial a_6}\right)\left(\frac{\partial a_6}{\partial z_6}\right)\left(\frac{\partial z_6}{\partial w_{21}}\right) \quad \text{where} \left(\frac{\partial z_6}{\partial w_{21}}\right) = a_1 \;\&\; \left(\frac{\partial a_6}{\partial z_6}\right) = \sigma'(z_6) = \frac{e^{z_6}}{(1+e^{z_6})^2} \quad \text{Equation S4.4b}$$

$$\left(\frac{\partial C}{\partial w_{22}}\right) = \left(\frac{\partial C}{\partial a_6}\right)\left(\frac{\partial a_6}{\partial z_6}\right)\left(\frac{\partial z_6}{\partial w_{22}}\right) \quad \text{where} \left(\frac{\partial z_6}{\partial w_{22}}\right) = a_2 \quad \text{Equation S4.4c}$$

$$\left(\frac{\partial C}{\partial w_{23}}\right) = \left(\frac{\partial C}{\partial a_6}\right)\left(\frac{\partial a_6}{\partial z_6}\right)\left(\frac{\partial z_6}{\partial w_{23}}\right) \quad \text{where} \left(\frac{\partial z_4}{\partial w_{23}}\right) = a_3 \quad \text{Equation S4.4d}$$

$\left(\frac{\partial C}{\partial w_{24}}\right) = \left(\frac{\partial C}{\partial a_6}\right)\left(\frac{\partial a_6}{\partial z_6}\right)\left(\frac{\partial z_6}{\partial w_{24}}\right)$ where $\left(\frac{\partial z_6}{\partial w_{24}}\right) = a_4$ Equation S4.4e

$\left(\frac{\partial C}{\partial w_{25}}\right) = \left(\frac{\partial C}{\partial a_6}\right)\left(\frac{\partial a_6}{\partial z_6}\right)\left(\frac{\partial z_6}{\partial w_{25}}\right)$ where $\left(\frac{\partial z_6}{\partial w_{25}}\right) = a_5$ Equation S4.4f

Hence: $\Delta b_6 = -L\left(\sum_{i=1}^{n}\left(\frac{\partial C}{\partial b_6}\right)\right)/n$ & $b_{6(New)} = b_{6(Old)} + \Delta b_6$ Equation S4.5a

$\Delta w_{21} = -L\left(\sum_{i=1}^{n}\left(\frac{\partial C}{\partial w_{21}}\right)\right)/n$ & $w_{21(New)} = w_{21(Old)} + \Delta w_{21}$ Equation S4.5b

$\Delta w_{22} = -L\left(\sum_{i=1}^{n}\left(\frac{\partial C}{\partial w_{22}}\right)\right)/n$ & $w_{22(New)} = w_{22(Old)} + \Delta w_{22}$ Equation S4.5c

$\Delta w_{23} = -L\left(\sum_{i=1}^{n}\left(\frac{\partial C}{\partial w_{23}}\right)\right)/n$ & $w_{23(New)} = w_{23(Old)} + \Delta w_{23}$ Equation S4.5d

$\Delta w_{24} = -L\left(\sum_{i=1}^{n}\left(\frac{\partial C}{\partial w_{24}}\right)\right)/n$ & $w_{24(New)} = w_{24(Old)} + \Delta w_{24}$ Equation S4.5e

$\Delta w_{25} = -L\left(\sum_{i=1}^{n}\left(\frac{\partial C}{\partial w_{25}}\right)\right)/n$ & $w_{25(New)} = w_{25(Old)} + \Delta w_{25}$ Equation S4.5f

$\left(\frac{\partial C}{\partial b_7}\right) = \left(\frac{\partial C}{\partial a_7}\right)\left(\frac{\partial a_7}{\partial z_7}\right)\left(\frac{\partial z_7}{\partial b_7}\right)$ where $\left(\frac{\partial z_7}{\partial b_7}\right) = 1$ Equation S4.6a

$\left(\frac{\partial C}{\partial w_{26}}\right) = \left(\frac{\partial C}{\partial a_7}\right)\left(\frac{\partial a_7}{\partial z_7}\right)\left(\frac{\partial z_7}{\partial w_{26}}\right)$ where $\left(\frac{\partial z_7}{\partial w_{26}}\right) = a_1$ & $\left(\frac{\partial a_7}{\partial z_7}\right) = \sigma'(z_7) = \frac{e^{z_7}}{(1+e^{z_7})^2}$ Equation S4.6b

$\left(\frac{\partial C}{\partial w_{27}}\right) = \left(\frac{\partial C}{\partial a_7}\right)\left(\frac{\partial a_7}{\partial z_7}\right)\left(\frac{\partial z_7}{\partial w_{27}}\right)$ where $\left(\frac{\partial z_7}{\partial w_{27}}\right) = a_2$ Equation S4.6c

$\left(\frac{\partial C}{\partial w_{28}}\right) = \left(\frac{\partial C}{\partial a_7}\right)\left(\frac{\partial a_7}{\partial z_7}\right)\left(\frac{\partial z_7}{\partial w_{28}}\right)$ where $\left(\frac{\partial z_7}{\partial w_{28}}\right) = a_3$ Equation S4.6d

$\left(\frac{\partial C}{\partial w_{29}}\right) = \left(\frac{\partial C}{\partial a_7}\right)\left(\frac{\partial a_7}{\partial z_7}\right)\left(\frac{\partial z_7}{\partial w_{29}}\right)$ where $\left(\frac{\partial z_7}{\partial w_{29}}\right) = a_4$ Equation S4.6e

$\left(\frac{\partial C}{\partial w_{30}}\right) = \left(\frac{\partial C}{\partial a_7}\right)\left(\frac{\partial a_7}{\partial z_7}\right)\left(\frac{\partial z_7}{\partial w_{30}}\right)$ where $\left(\frac{\partial z_7}{\partial w_{30}}\right) = a_5$ Equation S4.6f

Hence: $\Delta b_7 = -L\left(\sum_{i=1}^{n}\left(\frac{\partial C}{\partial b_7}\right)\right)/n$ & $b_{7(New)} = b_{7(Old)} + \Delta b_7$ Equation S4.7a

$\Delta w_{26} = -L\left(\sum_{i=1}^{n}\left(\frac{\partial C}{\partial w_{26}}\right)\right)/n$ & $w_{26(New)} = w_{26(Old)} + \Delta w_{26}$ Equation S4.7b

$\Delta w_{27} = -L\left(\sum_{i=1}^{n}\left(\frac{\partial C}{\partial w_{27}}\right)\right)/n$ & $w_{27(New)} = w_{27(Old)} + \Delta w_{27}$ Equation S4.7c

$\Delta w_{28} = -L\left(\sum_{i=1}^{n}\left(\frac{\partial C}{\partial w_{28}}\right)\right)/n$ & $w_{28(New)} = w_{28(Old)} + \Delta w_{28}$ Equation S4.7d

$\Delta w_{29} = -L\left(\sum_{i=1}^{n}\left(\frac{\partial C}{\partial w_{29}}\right)\right)/n$ & $w_{29(New)} = w_{29(Old)} + \Delta w_{29}$ Equation S4.7e

$\Delta w_{30} = -L\left(\sum_{i=1}^{n}\left(\frac{\partial C}{\partial w_{30}}\right)\right)/n$ & $w_{30(New)} = w_{30(Old)} + \Delta w_{30}$ Equation S4.7f

$\left(\frac{\partial C}{\partial b_8}\right) = \left(\frac{\partial C}{\partial a_8}\right)\left(\frac{\partial a_8}{\partial z_8}\right)\left(\frac{\partial z_8}{\partial b_8}\right)$ where $\left(\frac{\partial z_8}{\partial b_8}\right) = 1$ Equation S4.8a

$\left(\frac{\partial C}{\partial w_{31}}\right) = \left(\frac{\partial C}{\partial a_8}\right)\left(\frac{\partial a_8}{\partial z_8}\right)\left(\frac{\partial z_8}{\partial w_{31}}\right)$ where $\left(\frac{\partial z_8}{\partial w_{31}}\right) = a_1$ & $\left(\frac{\partial a_8}{\partial z_8}\right) = \sigma'(z_8) = \frac{e^{z_8}}{(1+e^{z_8})^2}$ Equation S4.8b

$\left(\frac{\partial C}{\partial w_{32}}\right) = \left(\frac{\partial C}{\partial a_8}\right)\left(\frac{\partial a_8}{\partial z_8}\right)\left(\frac{\partial z_8}{\partial w_{32}}\right)$ where $\left(\frac{\partial z_8}{\partial w_{32}}\right) = a_2$ Equation S4.9c

$\left(\frac{\partial C}{\partial w_{33}}\right) = \left(\frac{\partial C}{\partial a_8}\right)\left(\frac{\partial a_8}{\partial z_8}\right)\left(\frac{\partial z_8}{\partial w_{33}}\right)$ where $\left(\frac{\partial z_8}{\partial w_{33}}\right) = a_3$ Equation S4.9d

$\left(\frac{\partial C}{\partial w_{34}}\right) = \left(\frac{\partial C}{\partial a_8}\right)\left(\frac{\partial a_8}{\partial z_8}\right)\left(\frac{\partial z_8}{\partial w_{34}}\right)$ where $\left(\frac{\partial z_8}{\partial w_{34}}\right) = a_4$ Equation S4.9e

$$\left(\frac{\partial C}{\partial w_{35}}\right) = \left(\frac{\partial C}{\partial a_8}\right)\left(\frac{\partial a_8}{\partial z_8}\right)\left(\frac{\partial z_8}{\partial w_{35}}\right) \quad \text{where } \left(\frac{\partial z_8}{\partial w_{35}}\right) = a_5 \qquad \text{Equation S4.9f}$$

Hence: $\Delta b_8 = -L\left(\sum_{i=1}^{n}\left(\frac{\partial C}{\partial b_8}\right)\right)/n \;\;\&\;\; b_{8(New)} = b_{8(Old)} + \Delta b_8$ Equation S5.0a

$$\Delta w_{31} = -L\left(\sum_{i=1}^{n}\left(\frac{\partial C}{\partial w_{31}}\right)\right)/n \;\;\&\; w_{31(New)} = w_{31(Old)} + \Delta w_{31} \qquad \text{Equation S5.0b}$$

$$\Delta w_{32} = -L\left(\sum_{i=1}^{n}\left(\frac{\partial C}{\partial w_{32}}\right)\right)/n \;\;\&\; w_{32(New)} = w_{32(Old)} + \Delta w_{32} \qquad \text{Equation S5.0c}$$

$$\Delta w_{33} = -L\left(\sum_{i=1}^{n}\left(\frac{\partial C}{\partial w_{33}}\right)\right)/n \;\;\&\; w_{33(New)} = w_{33(Old)} + \Delta w_{33} \qquad \text{Equation S5.0d}$$

$$\Delta w_{34} = -L\left(\sum_{i=1}^{n}\left(\frac{\partial C}{\partial w_{34}}\right)\right)/n \;\;\&\; w_{34(New)} = w_{34(Old)} + \Delta w_{34} \qquad \text{Equation S5.0e}$$

$$\Delta w_{35} = -L\left(\sum_{i=1}^{n}\left(\frac{\partial C}{\partial w_{35}}\right)\right)/n \;\;\&\; w_{35(New)} = w_{35(Old)} + \Delta w_{35} \qquad \text{Equation S5.0f}$$

$$\left(\frac{\partial C}{\partial b_9}\right) = \left(\frac{\partial C}{\partial a_9}\right)\left(\frac{\partial a_9}{\partial z_9}\right)\left(\frac{\partial z_9}{\partial b_9}\right) \quad \text{where } \left(\frac{\partial z_9}{\partial b_9}\right) = 1 \qquad \text{Equation S5.1a}$$

$$\left(\frac{\partial C}{\partial w_{36}}\right) = \left(\frac{\partial C}{\partial a_9}\right)\left(\frac{\partial a_9}{\partial z_9}\right)\left(\frac{\partial z_9}{\partial w_{36}}\right) \quad \text{where } \left(\frac{\partial z_9}{\partial w_{36}}\right) = a_1 \;\&\; \left(\frac{\partial a_9}{\partial z_9}\right) = \sigma'(z_9) = \frac{e^{z_9}}{(1+e^{z_9})^2} \qquad \text{Equation S5.1b}$$

$$\left(\frac{\partial C}{\partial w_{37}}\right) = \left(\frac{\partial C}{\partial a_9}\right)\left(\frac{\partial a_9}{\partial z_9}\right)\left(\frac{\partial z_9}{\partial w_{37}}\right) \quad \text{where } \left(\frac{\partial z_9}{\partial w_{37}}\right) = a_2 \qquad \text{Equation S5.1c}$$

$$\left(\frac{\partial C}{\partial w_{38}}\right) = \left(\frac{\partial C}{\partial a_9}\right)\left(\frac{\partial a_9}{\partial z_9}\right)\left(\frac{\partial z_9}{\partial w_{38}}\right) \quad \text{where } \left(\frac{\partial z_9}{\partial w_{38}}\right) = a_3 \qquad \text{Equation S5.1d}$$

$$\left(\frac{\partial C}{\partial w_{39}}\right) = \left(\frac{\partial C}{\partial a_9}\right)\left(\frac{\partial a_9}{\partial z_9}\right)\left(\frac{\partial z_9}{\partial w_{39}}\right) \quad \text{where } \left(\frac{\partial z_9}{\partial w_{39}}\right) = a_4 \qquad \text{Equation S5.1e}$$

$$\left(\frac{\partial C}{\partial w_{40}}\right) = \left(\frac{\partial C}{\partial a_9}\right)\left(\frac{\partial a_9}{\partial z_9}\right)\left(\frac{\partial z_9}{\partial w_{40}}\right) \quad \text{where } \left(\frac{\partial z_9}{\partial w_{40}}\right) = a_5 \qquad \text{Equation S5.1f}$$

Hence: $\Delta b_9 = -L\left(\sum_{i=1}^{n}\left(\frac{\partial C}{\partial b_9}\right)\right)/n \;\;\&\;\; b_{9(New)} = b_{9(Old)} + \Delta b_9$ Equation S5.2a

$$\Delta w_{36} = -L\left(\sum_{i=1}^{n}\left(\frac{\partial C}{\partial w_{36}}\right)\right)/n \;\;\&\; w_{36(New)} = w_{36(Old)} + \Delta w_{36} \qquad \text{Equation S5.3b}$$

$$\Delta w_{37} = -L\left(\sum_{i=1}^{n}\left(\frac{\partial C}{\partial w_{37}}\right)\right)/n \;\;\&\; w_{37(New)} = w_{37(Old)} + \Delta w_{37} \qquad \text{Equation S5.3c}$$

$$\Delta w_{38} = -L\left(\sum_{i=1}^{n}\left(\frac{\partial C}{\partial w_{38}}\right)\right)/n \;\;\&\; w_{38(New)} = w_{38(Old)} + \Delta w_{38} \qquad \text{Equation S5.3d}$$

$$\Delta w_{39} = -L\left(\sum_{i=1}^{n}\left(\frac{\partial C}{\partial w_{39}}\right)\right)/n \;\;\&\; w_{39(New)} = w_{39(Old)} + \Delta w_{39} \qquad \text{Equation S5.3e}$$

$$\Delta w_{40} = -L\left(\sum_{i=1}^{n}\left(\frac{\partial C}{\partial w_{40}}\right)\right)/n \;\;\&\; w_{40(New)} = w_{40(Old)} + \Delta w_{40} \qquad \text{Equation S5.3f}$$

$$\left(\frac{\partial C}{\partial b_{10}}\right) = \left(\frac{\partial C}{\partial a_{10}}\right)\left(\frac{\partial a_{10}}{\partial z_{10}}\right)\left(\frac{\partial z_{10}}{\partial b_{10}}\right) \quad \text{where } \left(\frac{\partial z_{10}}{\partial b_{10}}\right) = 1 \qquad \text{Equation S5.4a}$$

$$\left(\frac{\partial C}{\partial w_{41}}\right) = \left(\frac{\partial C}{\partial a_{10}}\right)\left(\frac{\partial a_{10}}{\partial z_{10}}\right)\left(\frac{\partial z_{10}}{\partial w_{41}}\right) \quad \text{where } \left(\frac{\partial z_{10}}{\partial w_{41}}\right) = a_1 \;\&\; \left(\frac{\partial a_{10}}{\partial z_{10}}\right) = \sigma'(z_{10}) = \frac{e^{z_{10}}}{(1+e^{z_{10}})^2} \qquad \text{Equation S5.4b}$$

$$\left(\frac{\partial C}{\partial w_{42}}\right) = \left(\frac{\partial C}{\partial a_{10}}\right)\left(\frac{\partial a_{10}}{\partial z_{10}}\right)\left(\frac{\partial z_{10}}{\partial w_{42}}\right) \quad \text{where } \left(\frac{\partial z_{10}}{\partial w_{42}}\right) = a_2 \qquad \text{Equation S5.4c}$$

$$\left(\frac{\partial C}{\partial w_{43}}\right) = \left(\frac{\partial C}{\partial a_{10}}\right)\left(\frac{\partial a_{10}}{\partial z_{10}}\right)\left(\frac{\partial z_{10}}{\partial w_{43}}\right) \quad \text{where } \left(\frac{\partial z_{10}}{\partial w_{43}}\right) = a_3 \qquad \text{Equation S5.4d}$$

$$\left(\frac{\partial C}{\partial w_{44}}\right) = \left(\frac{\partial C}{\partial a_{10}}\right)\left(\frac{\partial a_{10}}{\partial z_{10}}\right)\left(\frac{\partial z_{10}}{\partial w_{44}}\right) \quad \text{where } \left(\frac{\partial z_{10}}{\partial w_{44}}\right) = a_4 \qquad \text{Equation S5.4e}$$

$$\left(\frac{\partial C}{\partial w_{45}}\right) = \left(\frac{\partial C}{\partial a_{10}}\right)\left(\frac{\partial a_{10}}{\partial z_{10}}\right)\left(\frac{\partial z_{10}}{\partial w_{45}}\right) \quad \text{where } \left(\frac{\partial z_{10}}{\partial w_{45}}\right) = a_5 \qquad \text{Equation S5.4f}$$

Hence: $\Delta b_{10} = -L\left(\sum_{i=1}^{n}\left(\frac{\partial C}{\partial b_{10}}\right)\right)/n \;\;\&\;\; b_{10(New)} = b_{10(Old)} + \Delta b_{10}$ Equation S5.5a

$\Delta w_{41} = -L\left(\sum_{i=1}^{n}\left(\frac{\partial C}{\partial w_{41}}\right)\right)/n \quad \& \; w_{41(New)} = w_{41(Old)} + \Delta w_{41}$ Equation S5.5b

$\Delta w_{42} = -L\left(\sum_{i=1}^{n}\left(\frac{\partial C}{\partial w_{42}}\right)\right)/n \quad \& \; w_{42(New)} = w_{42(Old)} + \Delta w_{42}$ Equation S5.5c

$\Delta w_{43} = -L\left(\sum_{i=1}^{n}\left(\frac{\partial C}{\partial w_{43}}\right)\right)/n \quad \& \; w_{43(New)} = w_{43(Old)} + \Delta w_{43}$ Equation S5.5d

$\Delta w_{44} = -L\left(\sum_{i=1}^{n}\left(\frac{\partial C}{\partial w_{44}}\right)\right)/n \quad \& \; w_{44(New)} = w_{44(Old)} + \Delta w_{44}$ Equation S5.5e

$\Delta w_{45} = -L\left(\sum_{i=1}^{n}\left(\frac{\partial C}{\partial w_{45}}\right)\right)/n \quad \& \; w_{45(New)} = w_{45(Old)} + \Delta w_{45}$ Equation S5.5f

3. The overall cost function on hidden neurons (HNs) $a_1 - a_5$.

This stage of mathematical formulation requires setting up the overall cost function of the selected hidden neurons ($a_1 - a_5$) against the cost function of the preceding activation (a_i), cost function and sum weights. For instance: $\left(\frac{\partial C}{\partial a_f}\right) = \left(\frac{\partial C}{\partial a_i}\right)\left(\frac{\partial a_i}{\partial z}\right)\left(\frac{\partial z}{\partial a_f}\right)$ where $w_f = \left(\frac{\partial z}{\partial a_f}\right)$ Equation S5.6

Thus, the following mathematical models are obtained from the application of Equation S5.6 to the DNN framework in Figure S1.

$\left(\frac{\partial C}{\partial a_1}\right)_1 = \left(\frac{\partial C}{\partial a_6}\right)\left(\frac{\partial a_6}{\partial z_6}\right)\left(\frac{\partial z_6}{\partial a_1}\right)$ where $\left(\frac{\partial a_6}{\partial z_6}\right) = \sigma'(z_6) = \frac{e^{z_6}}{(1+e^{z_6})^2}$ & $\left(\frac{\partial z_6}{\partial a_1}\right) = w_{21}$ Equation S5.7a

$\left(\frac{\partial C}{\partial a_1}\right)_2 = \left(\frac{\partial C}{\partial a_7}\right)\left(\frac{\partial a_7}{\partial z_7}\right)\left(\frac{\partial z_7}{\partial a_1}\right)$ where $\left(\frac{\partial a_7}{\partial z_7}\right) = \sigma'(z_7) = \frac{e^{z_7}}{(1+e^{z_7})^2}$ & $\left(\frac{\partial z_7}{\partial a_1}\right) = w_{26}$ Equation S5.7b

$\left(\frac{\partial C}{\partial a_1}\right)_3 = \left(\frac{\partial C}{\partial a_8}\right)\left(\frac{\partial a_8}{\partial z_8}\right)\left(\frac{\partial z_8}{\partial a_1}\right)$ where $\left(\frac{\partial a_8}{\partial z_8}\right) = \sigma'(z_8) = \frac{e^{z_8}}{(1+e^{z_8})^2}$ & $\left(\frac{\partial z_8}{\partial a_1}\right) = w_{31}$ Equation S5.7c

$\left(\frac{\partial C}{\partial a_1}\right)_4 = \left(\frac{\partial C}{\partial a_9}\right)\left(\frac{\partial a_9}{\partial z_9}\right)\left(\frac{\partial z_9}{\partial a_1}\right)$ where $\left(\frac{\partial a_9}{\partial z_9}\right) = \sigma'(z_9) = \frac{e^{z_9}}{(1+e^{z_9})^2}$ & $\left(\frac{\partial z_9}{\partial a_1}\right) = w_{36}$ Equation S5.7d

$\left(\frac{\partial C}{\partial a_1}\right)_5 = \left(\frac{\partial C}{\partial a_{10}}\right)\left(\frac{\partial a_{10}}{\partial z_{10}}\right)\left(\frac{\partial z_{10}}{\partial a_1}\right)$ where $\left(\frac{\partial a_{10}}{\partial z_{10}}\right) = \sigma'(z_{10}) = \frac{e^{z_{10}}}{(1+e^{z_{10}})^2}$ & $\left(\frac{\partial z_{10}}{\partial a_1}\right) = w_{41}$ Equation S5.7e

$\left(\frac{\partial C}{\partial a_1}\right) = \left(\frac{\partial C}{\partial a_1}\right)_1 + \left(\frac{\partial C}{\partial a_1}\right)_2 + \left(\frac{\partial C}{\partial a_1}\right)_3 + \left(\frac{\partial C}{\partial a_1}\right)_4 + \left(\frac{\partial C}{\partial a_1}\right)_5$ Equation S5.7f

$\left(\frac{\partial C}{\partial a_2}\right)_1 = \left(\frac{\partial C}{\partial a_6}\right)\left(\frac{\partial a_6}{\partial z_6}\right)\left(\frac{\partial z_6}{\partial a_2}\right)$ where $\left(\frac{\partial a_6}{\partial z_6}\right) = \sigma'(z_6) = \frac{e^{z_6}}{(1+e^{z_6})^2}$ & $\left(\frac{\partial z_6}{\partial a_2}\right) = w_{22}$ Equation S5.8a

$\left(\frac{\partial C}{\partial a_2}\right)_2 = \left(\frac{\partial C}{\partial a_7}\right)\left(\frac{\partial a_7}{\partial z_7}\right)\left(\frac{\partial z_7}{\partial a_2}\right)$ where $\left(\frac{\partial a_7}{\partial z_7}\right) = \sigma'(z_7) = \frac{e^{z_7}}{(1+e^{z_7})^2}$ & $\left(\frac{\partial z_7}{\partial a_2}\right) = w_{27}$ Equation S5.8b

$\left(\frac{\partial C}{\partial a_2}\right)_3 = \left(\frac{\partial C}{\partial a_8}\right)\left(\frac{\partial a_8}{\partial z_8}\right)\left(\frac{\partial z_8}{\partial a_2}\right)$ where $\left(\frac{\partial a_8}{\partial z_8}\right) = \sigma'(z_8) = \frac{e^{z_8}}{(1+e^{z_8})^2}$ & $\left(\frac{\partial z_8}{\partial a_2}\right) = w_{32}$ Equation S5.8c

$\left(\frac{\partial C}{\partial a_2}\right)_4 = \left(\frac{\partial C}{\partial a_9}\right)\left(\frac{\partial a_9}{\partial z_9}\right)\left(\frac{\partial z_9}{\partial a_2}\right)$ where $\left(\frac{\partial a_9}{\partial z_9}\right) = \sigma'(z_9) = \frac{e^{z_9}}{(1+e^{z_9})^2}$ & $\left(\frac{\partial z_9}{\partial a_2}\right) = w_{37}$ Equation S5.8d

$\left(\frac{\partial C}{\partial a_2}\right)_5 = \left(\frac{\partial C}{\partial a_{10}}\right)\left(\frac{\partial a_{10}}{\partial z_{10}}\right)\left(\frac{\partial z_{10}}{\partial a_2}\right)$ where $\left(\frac{\partial a_{10}}{\partial z_{10}}\right) = \sigma'(z_{10}) = \frac{e^{z_{10}}}{(1+e^{z_{10}})^2}$ & $\left(\frac{\partial z_{10}}{\partial a_2}\right) = w_{42}$ Equation S5.8e

$\left(\frac{\partial C}{\partial a_2}\right) = \left(\frac{\partial C}{\partial a_2}\right)_1 + \left(\frac{\partial C}{\partial a_2}\right)_2 + \left(\frac{\partial C}{\partial a_2}\right)_3 + \left(\frac{\partial C}{\partial a_2}\right)_4 + \left(\frac{\partial C}{\partial a_2}\right)_5$ Equation S5.8f

$\left(\frac{\partial C}{\partial a_3}\right)_1 = \left(\frac{\partial C}{\partial a_6}\right)\left(\frac{\partial a_6}{\partial z_6}\right)\left(\frac{\partial z_6}{\partial a_3}\right)$ where $\left(\frac{\partial a_6}{\partial z_6}\right) = \sigma'(z_6) = \frac{e^{z_6}}{(1+e^{z_6})^2}$ & $\left(\frac{\partial z_6}{\partial a_3}\right) = w_{23}$ Equation S5.9a

$\left(\frac{\partial C}{\partial a_3}\right)_2 = \left(\frac{\partial C}{\partial a_7}\right)\left(\frac{\partial a_7}{\partial z_7}\right)\left(\frac{\partial z_7}{\partial a_3}\right)$ where $\left(\frac{\partial a_7}{\partial z_7}\right) = \sigma'(z_7) = \frac{e^{z_7}}{(1+e^{z_7})^2}$ & $\left(\frac{\partial z_7}{\partial a_3}\right) = w_{28}$ Equation S5.9b

$\left(\frac{\partial C}{\partial a_3}\right)_3 = \left(\frac{\partial C}{\partial a_8}\right)\left(\frac{\partial a_8}{\partial z_8}\right)\left(\frac{\partial z_8}{\partial a_3}\right)$ where $\left(\frac{\partial a_8}{\partial z_8}\right) = \sigma'(z_8) = \frac{e^{z_8}}{(1+e^{z_8})^2}$ & $\left(\frac{\partial z_8}{\partial a_3}\right) = w_{33}$ Equation S5.9c

$\left(\frac{\partial C}{\partial a_3}\right)_4 = \left(\frac{\partial C}{\partial a_9}\right)\left(\frac{\partial a_9}{\partial z_9}\right)\left(\frac{\partial z_9}{\partial a_3}\right)$ where $\left(\frac{\partial a_9}{\partial z_9}\right) = \sigma'(z_9) = \frac{e^{z_9}}{(1+e^{z_9})^2}$ & $\left(\frac{\partial z_9}{\partial a_3}\right) = w_{38}$ Equation S5.9d

$\left(\frac{\partial C}{\partial a_3}\right)_5 = \left(\frac{\partial C}{\partial a_{10}}\right)\left(\frac{\partial a_{10}}{\partial z_{10}}\right)\left(\frac{\partial z_{10}}{\partial a_3}\right)$ where $\left(\frac{\partial a_{10}}{\partial z_{10}}\right) = \sigma'(z_{10}) = \frac{e^{z_{10}}}{(1+e^{z_{10}})^2}$ & $\left(\frac{\partial z_{10}}{\partial a_3}\right) = w_{43}$ Equation S5.9e

$\left(\frac{\partial C}{\partial a_3}\right) = \left(\frac{\partial C}{\partial a_3}\right)_1 + \left(\frac{\partial C}{\partial a_3}\right)_2 + \left(\frac{\partial C}{\partial a_3}\right)_3 + \left(\frac{\partial C}{\partial a_3}\right)_4 + \left(\frac{\partial C}{\partial a_3}\right)_5$ Equation S5.9f

$\left(\frac{\partial C}{\partial a_4}\right)_1 = \left(\frac{\partial C}{\partial a_6}\right)\left(\frac{\partial a_6}{\partial z_6}\right)\left(\frac{\partial z_6}{\partial a_4}\right)$ where $\left(\frac{\partial a_6}{\partial z_6}\right) = \sigma'(z_6) = \frac{e^{z_6}}{(1+e^{z_6})^2}$ & $\left(\frac{\partial z_6}{\partial a_4}\right) = w_{24}$ Equation S6.0a

$\left(\frac{\partial C}{\partial a_4}\right)_2 = \left(\frac{\partial C}{\partial a_7}\right)\left(\frac{\partial a_7}{\partial z_7}\right)\left(\frac{\partial z_7}{\partial a_4}\right)$ where $\left(\frac{\partial a_7}{\partial z_7}\right) = \sigma'(z_7) = \frac{e^{z_7}}{(1+e^{z_7})^2}$ & $\left(\frac{\partial z_7}{\partial a_4}\right) = w_{29}$ Equation S6.0b

$\left(\frac{\partial C}{\partial a_4}\right)_3 = \left(\frac{\partial C}{\partial a_8}\right)\left(\frac{\partial a_8}{\partial z_8}\right)\left(\frac{\partial z_8}{\partial a_4}\right)$ where $\left(\frac{\partial a_8}{\partial z_8}\right) = \sigma'(z_8) = \frac{e^{z_8}}{(1+e^{z_8})^2}$ & $\left(\frac{\partial z_8}{\partial a_4}\right) = w_{34}$ Equation S6.0c

$\left(\frac{\partial C}{\partial a_4}\right)_4 = \left(\frac{\partial C}{\partial a_9}\right)\left(\frac{\partial a_9}{\partial z_9}\right)\left(\frac{\partial z_9}{\partial a_4}\right)$ where $\left(\frac{\partial a_9}{\partial z_9}\right) = \sigma'(z_9) = \frac{e^{z_9}}{(1+e^{z_9})^2}$ & $\left(\frac{\partial z_9}{\partial a_4}\right) = w_{39}$ Equation S6.0d

$\left(\frac{\partial C}{\partial a_4}\right)_5 = \left(\frac{\partial C}{\partial a_{10}}\right)\left(\frac{\partial a_{10}}{\partial z_{10}}\right)\left(\frac{\partial z_{10}}{\partial a_4}\right)$ where $\left(\frac{\partial a_{10}}{\partial z_{10}}\right) = \sigma'(z_{10}) = \frac{e^{z_{10}}}{(1+e^{z_{10}})^2}$ & $\left(\frac{\partial z_{10}}{\partial a_4}\right) = w_{44}$ Equation S6.0e

$\left(\frac{\partial C}{\partial a_4}\right) = \left(\frac{\partial C}{\partial a_4}\right)_1 + \left(\frac{\partial C}{\partial a_4}\right)_2 + \left(\frac{\partial C}{\partial a_4}\right)_3 + \left(\frac{\partial C}{\partial a_4}\right)_4 + \left(\frac{\partial C}{\partial a_4}\right)_5$ Equation S6.0f

$\left(\frac{\partial C}{\partial a_5}\right)_1 = \left(\frac{\partial C}{\partial a_6}\right)\left(\frac{\partial a_6}{\partial z_6}\right)\left(\frac{\partial z_6}{\partial a_5}\right)$ where $\left(\frac{\partial a_6}{\partial z_6}\right) = \sigma'(z_6) = \frac{e^{z_6}}{(1+e^{z_6})^2}$ & $\left(\frac{\partial z_6}{\partial a_5}\right) = w_{25}$ Equation S6.1a

$\left(\frac{\partial C}{\partial a_5}\right)_2 = \left(\frac{\partial C}{\partial a_7}\right)\left(\frac{\partial a_7}{\partial z_7}\right)\left(\frac{\partial z_7}{\partial a_5}\right)$ where $\left(\frac{\partial a_7}{\partial z_7}\right) = \sigma'(z_7) = \frac{e^{z_7}}{(1+e^{z_7})^2}$ & $\left(\frac{\partial z_7}{\partial a_5}\right) = w_{30}$ Equation S6.1b

$\left(\frac{\partial C}{\partial a_5}\right)_3 = \left(\frac{\partial C}{\partial a_8}\right)\left(\frac{\partial a_8}{\partial z_8}\right)\left(\frac{\partial z_8}{\partial a_5}\right)$ where $\left(\frac{\partial a_8}{\partial z_8}\right) = \sigma'(z_8) = \frac{e^{z_8}}{(1+e^{z_8})^2}$ & $\left(\frac{\partial z_8}{\partial a_5}\right) = w_{35}$ Equation S6.1c

$\left(\frac{\partial C}{\partial a_5}\right)_4 = \left(\frac{\partial C}{\partial a_9}\right)\left(\frac{\partial a_9}{\partial z_9}\right)\left(\frac{\partial z_9}{\partial a_5}\right)$ where $\left(\frac{\partial a_9}{\partial z_9}\right) = \sigma'(z_9) = \frac{e^{z_9}}{(1+e^{z_9})^2}$ & $\left(\frac{\partial z_9}{\partial a_5}\right) = w_{40}$ Equation S6.1d

$\left(\frac{\partial C}{\partial a_5}\right)_5 = \left(\frac{\partial C}{\partial a_{10}}\right)\left(\frac{\partial a_{10}}{\partial z_{10}}\right)\left(\frac{\partial z_{10}}{\partial a_5}\right)$ where $\left(\frac{\partial a_{10}}{\partial z_{10}}\right) = \sigma'(z_{10}) = \frac{e^{z_{10}}}{(1+e^{z_{10}})^2}$ & $\left(\frac{\partial z_{10}}{\partial a_5}\right) = w_{45}$ Equation S6.1e

$\left(\frac{\partial C}{\partial a_5}\right) = \left(\frac{\partial C}{\partial a_5}\right)_1 + \left(\frac{\partial C}{\partial a_5}\right)_2 + \left(\frac{\partial C}{\partial a_5}\right)_3 + \left(\frac{\partial C}{\partial a_5}\right)_4 + \left(\frac{\partial C}{\partial a_5}\right)_5$ Equation S6.1f

4. The overall cost function on the input neurons $x_1 - x_5$.

This is the last stage of the cost optimisation techniques, and this approach requires going back to the familiar territory of adopting the single neural network of formulating mathematical models for the estimation of the changes in biases and weights connecting the input functions to the hidden neurons $a_1 - a_5$.

$\left(\frac{\partial C}{\partial b_1}\right) = \left(\frac{\partial C}{\partial a_1}\right)\left(\frac{\partial a_1}{\partial z_1}\right)\left(\frac{\partial z_1}{\partial b_1}\right)$ where $\left(\frac{\partial z_1}{\partial b_1}\right) = 1$ Equation S6.2a

$\left(\frac{\partial C}{\partial w_1}\right) = \left(\frac{\partial C}{\partial a_1}\right)\left(\frac{\partial a_1}{\partial z_1}\right)\left(\frac{\partial z_1}{\partial w_1}\right)$ where $\left(\frac{\partial z_1}{\partial w_1}\right) = x_1$ & $\left(\frac{\partial a_1}{\partial z_1}\right) = \sigma'(z_1) = \frac{e^{z_1}}{(1+e^{z_1})^2}$ Equation S6.2b

$\left(\frac{\partial C}{\partial w_2}\right) = \left(\frac{\partial C}{\partial a_1}\right)\left(\frac{\partial a_1}{\partial z_1}\right)\left(\frac{\partial z_1}{\partial w_2}\right)$ where $\left(\frac{\partial z_1}{\partial w_2}\right) = x_2$ Equation S6.2c

$\left(\frac{\partial C}{\partial w_3}\right) = \left(\frac{\partial C}{\partial a_1}\right)\left(\frac{\partial a_1}{\partial z_1}\right)\left(\frac{\partial z_1}{\partial w_3}\right)$ where $\left(\frac{\partial z_1}{\partial w_3}\right) = x_3$ Equation S6.2d

$\left(\frac{\partial C}{\partial w_4}\right) = \left(\frac{\partial C}{\partial a_1}\right)\left(\frac{\partial a_1}{\partial z_1}\right)\left(\frac{\partial z_1}{\partial w_4}\right)$ where $\left(\frac{\partial z_1}{\partial w_4}\right) = x_4$ Equation S6.2e

Hence: $\Delta b_1 = -L\left(\sum_{i=1}^{n}\left(\frac{\partial C}{\partial b_1}\right)\right)/n$ & $b_{1(Old)} = b_{1(New)} + \Delta b_1$ Equation S6.3a

$\Delta w_1 = -L\left(\sum_{i=1}^{n}\left(\frac{\partial C}{\partial w_1}\right)\right)/n$ & $w_{1(New)} = w_{1(Old)} + \Delta w_1$ Equation S6.3b

$\Delta w_2 = -L\left(\sum_{i=1}^{n}\left(\frac{\partial C}{\partial w_2}\right)\right)/n$ & $w_{2(New)} = w_{2(Old)} + \Delta w_2$ Equation S6.3c

$\Delta w_3 = -L\left(\sum_{i=1}^{n}\left(\frac{\partial C}{\partial w_3}\right)\right)/n$ & $w_{3(New)} = w_{3(Old)} + \Delta w_3$ Equation S6.3d

$\Delta w_4 = -L\left(\sum_{i=1}^{n}\left(\frac{\partial C}{\partial w_4}\right)\right)/n$ & $w_{4(New)} = w_{4(Old)} + \Delta w_4$ Equation S6.3e

$\left(\frac{\partial C}{\partial b_2}\right) = \left(\frac{\partial C}{\partial a_2}\right)\left(\frac{\partial a_2}{\partial z_2}\right)\left(\frac{\partial z_2}{\partial b_2}\right)$ where $\left(\frac{\partial z_2}{\partial b_2}\right) = 1$ Equation S6.4a

$\left(\frac{\partial C}{\partial w_5}\right) = \left(\frac{\partial C}{\partial a_2}\right)\left(\frac{\partial a_2}{\partial z_2}\right)\left(\frac{\partial z_2}{\partial w_5}\right)$ where $\left(\frac{\partial z_2}{\partial w_5}\right) = x_1$ & $\left(\frac{\partial a_2}{\partial z_2}\right) = \sigma'(z_2) = \frac{e^{z_2}}{(1+e^{z_2})^2}$ Equation S6.4b

$\left(\frac{\partial C}{\partial w_6}\right) = \left(\frac{\partial C}{\partial a_2}\right)\left(\frac{\partial a_2}{\partial z_2}\right)\left(\frac{\partial z_2}{\partial w_6}\right)$ where $\left(\frac{\partial z_2}{\partial w_6}\right) = x_2$ Equation S6.4c

$\left(\frac{\partial C}{\partial w_7}\right) = \left(\frac{\partial C}{\partial a_2}\right)\left(\frac{\partial a_2}{\partial z_2}\right)\left(\frac{\partial z_2}{\partial w_7}\right)$ where $\left(\frac{\partial z_2}{\partial w_7}\right) = x_3$ Equation S6.4d

$\left(\frac{\partial C}{\partial w_8}\right) = \left(\frac{\partial C}{\partial a_2}\right)\left(\frac{\partial a_2}{\partial z_2}\right)\left(\frac{\partial z_2}{\partial w_8}\right)$ where $\left(\frac{\partial z_2}{\partial w_8}\right) = x_4$ Equation S6.4e

Hence: $\Delta b_2 = -L\left(\sum_{i=1}^{n}\left(\frac{\partial C}{\partial b_2}\right)\right)/n$ & $b_{2(Old)} = b_{2(New)} + \Delta b_2$ Equation S6.5a

$\Delta w_5 = -L\left(\sum_{i=1}^{n}\left(\frac{\partial C}{\partial w_5}\right)\right)/n$ & $w_{5(New)} = w_{5(Old)} + \Delta w_5$ Equation S6.5b

$\Delta w_6 = -L\left(\sum_{i=1}^{n}\left(\frac{\partial C}{\partial w_6}\right)\right)/n$ & $w_{6(New)} = w_{6(Old)} + \Delta w_6$ Equation S6.5c

$\Delta w_7 = -L\left(\sum_{i=1}^{n}\left(\frac{\partial C}{\partial w_7}\right)\right)/n$ & $w_{7(New)} = w_{7(Old)} + \Delta w_7$ Equation S6.5d

$\Delta w_8 = -L\left(\sum_{i=1}^{n}\left(\frac{\partial C}{\partial w_8}\right)\right)/n$ & $w_{8(New)} = w_{8(Old)} + \Delta w_8$ Equation S6.5e

$\left(\frac{\partial C}{\partial b_3}\right) = \left(\frac{\partial C}{\partial a_3}\right)\left(\frac{\partial a_3}{\partial z_3}\right)\left(\frac{\partial z_3}{\partial b_3}\right)$ where $\left(\frac{\partial z_3}{\partial b_3}\right) = 1$ Equation S6.6a

$\left(\frac{\partial C}{\partial w_9}\right) = \left(\frac{\partial C}{\partial a_3}\right)\left(\frac{\partial a_3}{\partial z_3}\right)\left(\frac{\partial z_3}{\partial w_9}\right)$ where $\left(\frac{\partial z_3}{\partial w_9}\right) = x_1$ & $\left(\frac{\partial a_3}{\partial z_3}\right) = \sigma'(z_3) = \frac{e^{z_3}}{(1+e^{z_3})^2}$ Equation S6.6b

$\left(\frac{\partial C}{\partial w_{10}}\right) = \left(\frac{\partial C}{\partial a_3}\right)\left(\frac{\partial a_3}{\partial z_3}\right)\left(\frac{\partial z_3}{\partial w_{10}}\right)$ where $\left(\frac{\partial z_3}{\partial w_{10}}\right) = x_2$ Equation S6.6c

$\left(\frac{\partial C}{\partial w_{11}}\right) = \left(\frac{\partial C}{\partial a_3}\right)\left(\frac{\partial a_3}{\partial z_3}\right)\left(\frac{\partial z_3}{\partial w_{11}}\right)$ where $\left(\frac{\partial z_3}{\partial w_{11}}\right) = x_3$ Equation S6.6d

$\left(\frac{\partial C}{\partial w_{12}}\right) = \left(\frac{\partial C}{\partial a_3}\right)\left(\frac{\partial a_3}{\partial z_3}\right)\left(\frac{\partial z_3}{\partial w_{12}}\right)$ where $\left(\frac{\partial z_3}{\partial w_{12}}\right) = x_4$ Equation S6.6e

Hence: $\Delta b_3 = -L\left(\sum_{i=1}^{n}\left(\frac{\partial C}{\partial b_3}\right)\right)/n$ & $b_{3(Old)} = b_{3(New)} + \Delta b_3$ Equation S6.7a

$$\Delta w_9 = -L\left(\sum_{i=1}^{n}\left(\frac{\partial C}{\partial w_9}\right)\right)/n \quad \& \; w_{9(New)} = w_{9(Old)} + \Delta w_9 \qquad \text{Equation S6.7b}$$

$$\Delta w_{10} = -L\left(\sum_{i=1}^{n}\left(\frac{\partial C}{\partial w_{10}}\right)\right)/n \quad \& \; w_{10(New)} = w_{10(Old)} + \Delta w_{10} \qquad \text{Equation S6.7c}$$

$$\Delta w_{11} = -L\left(\sum_{i=1}^{n}\left(\frac{\partial C}{\partial w_{11}}\right)\right)/n \quad \& \; w_{11(New)} = w_{11(Old)} + \Delta w_{11} \qquad \text{Equation S6.7d}$$

$$\Delta w_{12} = -L\left(\sum_{i=1}^{n}\left(\frac{\partial C}{\partial w_{12}}\right)\right)/n \quad \& \; w_{12(New)} = w_{12(Old)} + \Delta w_{12} \qquad \text{Equation S6.7e}$$

$$\left(\frac{\partial C}{\partial b_4}\right) = \left(\frac{\partial C}{\partial a_4}\right)\left(\frac{\partial a_4}{\partial z_4}\right)\left(\frac{\partial z_4}{\partial b_4}\right) \text{ where } \left(\frac{\partial z_4}{\partial b_4}\right) = 1 \qquad \text{Equation S6.8a}$$

$$\left(\frac{\partial C}{\partial w_{13}}\right) = \left(\frac{\partial C}{\partial a_4}\right)\left(\frac{\partial a_4}{\partial z_4}\right)\left(\frac{\partial z_4}{\partial w_{13}}\right) \text{ where } \left(\frac{\partial z_4}{\partial w_{13}}\right) = x_1 \; \& \left(\frac{\partial a_4}{\partial z_4}\right) = \sigma'(z_4) = \frac{e^{z_4}}{(1+e^{z_4})^2} \qquad \text{Equation S6.8b}$$

$$\left(\frac{\partial C}{\partial w_{14}}\right) = \left(\frac{\partial C}{\partial a_4}\right)\left(\frac{\partial a_4}{\partial z_4}\right)\left(\frac{\partial z_4}{\partial w_{14}}\right) \text{ where } \left(\frac{\partial z_4}{\partial w_{14}}\right) = x_2 \qquad \text{Equation S6.8c}$$

$$\left(\frac{\partial C}{\partial w_{15}}\right) = \left(\frac{\partial C}{\partial a_4}\right)\left(\frac{\partial a_4}{\partial z_4}\right)\left(\frac{\partial z_4}{\partial w_{15}}\right) \text{ where } \left(\frac{\partial z_4}{\partial w_{15}}\right) = x_3 \qquad \text{Equation S6.8d}$$

$$\left(\frac{\partial C}{\partial w_{16}}\right) = \left(\frac{\partial C}{\partial a_4}\right)\left(\frac{\partial a_4}{\partial z_4}\right)\left(\frac{\partial z_4}{\partial w_{16}}\right) \text{ where } \left(\frac{\partial z_4}{\partial w_{16}}\right) = x_4 \qquad \text{Equation S6.8e}$$

Hence: $\Delta b_4 = -L\left(\sum_{i=1}^{n}\left(\frac{\partial C}{\partial b_4}\right)\right)/n \quad \& \; b_{4(Old)} = b_{4(New)} + \Delta b_4$ Equation S6.9a

$$\Delta w_{13} = -L\left(\sum_{i=1}^{n}\left(\frac{\partial C}{\partial w_{13}}\right)\right)/n \quad \& \; w_{13(New)} = w_{13(Old)} + \Delta w_{13} \qquad \text{Equation S6.9b}$$

$$\Delta w_{14} = -L\left(\sum_{i=1}^{n}\left(\frac{\partial C}{\partial w_{14}}\right)\right)/n \quad \& \; w_{14(New)} = w_{14(Old)} + \Delta w_{14} \qquad \text{Equation S6.9c}$$

$$\Delta w_{15} = -L\left(\sum_{i=1}^{n}\left(\frac{\partial C}{\partial w_{15}}\right)\right)/n \quad \& \; w_{15(New)} = w_{15(Old)} + \Delta w_{15} \qquad \text{Equation S6.9d}$$

$$\Delta w_{16} = -L\left(\sum_{i=1}^{n}\left(\frac{\partial C}{\partial w_{16}}\right)\right)/n \quad \& \; w_{16(New)} = w_{16(Old)} + \Delta w_{16} \qquad \text{Equation S6.9e}$$

$$\left(\frac{\partial C}{\partial b_5}\right) = \left(\frac{\partial C}{\partial a_5}\right)\left(\frac{\partial a_5}{\partial z_5}\right)\left(\frac{\partial z_5}{\partial b_5}\right) \text{ where } \left(\frac{\partial z_5}{\partial b_5}\right) = 1 \qquad \text{Equation S7.0a}$$

$$\left(\frac{\partial C}{\partial w_{17}}\right) = \left(\frac{\partial C}{\partial a_5}\right)\left(\frac{\partial a_5}{\partial z_5}\right)\left(\frac{\partial z_5}{\partial w_{17}}\right) \text{ where } \left(\frac{\partial z_5}{\partial w_{17}}\right) = x_1 \; \& \left(\frac{\partial a_5}{\partial z_5}\right) = \sigma'(z_5) = \frac{e^{z_5}}{(1+e^{z_5})^2} \qquad \text{Equation S7.0b}$$

$$\left(\frac{\partial C}{\partial w_{18}}\right) = \left(\frac{\partial C}{\partial a_5}\right)\left(\frac{\partial a_5}{\partial z_5}\right)\left(\frac{\partial z_5}{\partial w_{18}}\right) \text{ where } \left(\frac{\partial z_5}{\partial w_{18}}\right) = x_2 \qquad \text{Equation S7.0c}$$

$$\left(\frac{\partial C}{\partial w_{19}}\right) = \left(\frac{\partial C}{\partial a_5}\right)\left(\frac{\partial a_5}{\partial z_5}\right)\left(\frac{\partial z_5}{\partial w_{19}}\right) \text{ where } \left(\frac{\partial z_5}{\partial w_{19}}\right) = x_3 \qquad \text{Equation S7.0d}$$

$$\left(\frac{\partial C}{\partial w_{20}}\right) = \left(\frac{\partial C}{\partial a_5}\right)\left(\frac{\partial a_5}{\partial z_5}\right)\left(\frac{\partial z_5}{\partial w_{20}}\right) \text{ where } \left(\frac{\partial z_5}{\partial w_{20}}\right) = x_4 \qquad \text{Equation S7.0e}$$

Hence: $\Delta b_5 = -L\left(\sum_{i=1}^{n}\left(\frac{\partial C}{\partial b_5}\right)\right)/n \quad \& \; b_{5(Old)} = b_{5(New)} + \Delta b_5$ Equation S7.1a

$$\Delta w_{17} = -L\left(\sum_{i=1}^{n}\left(\frac{\partial C}{\partial w_{17}}\right)\right)/n \quad \& \; w_{17(New)} = w_{17(Old)} + \Delta w_{17} \qquad \text{Equation S7.1b}$$

$$\Delta w_{18} = -L\left(\sum_{i=1}^{n}\left(\frac{\partial C}{\partial w_{18}}\right)\right)/n \quad \& \; w_{18(New)} = w_{18(Old)} + \Delta w_{18} \qquad \text{Equation S7.1c}$$

$$\Delta w_{19} = -L\left(\sum_{i=1}^{n}\left(\frac{\partial C}{\partial w_{19}}\right)\right)/n \quad \& \; w_{19(New)} = w_{19(Old)} + \Delta w_{19} \qquad \text{Equation S7.1d}$$

$$\Delta w_{20} = -L\left(\sum_{i=1}^{n}\left(\frac{\partial C}{\partial w_{20}}\right)\right)/n \quad \& \; w_{20(New)} = w_{20(Old)} + \Delta w_{20} \qquad \text{Equation S7.1e}$$

WRITTEN SCRIPT

```
Sub Hello()
    Range("R10:BZ10") = Range("R16:BZ16").Value
    Application.OnTime Now + TimeValue("00:00:01"), "Hello"
End Sub
```
